# METTL3 regulates breast cancer-associated alternative splicing switches

**DOI:** 10.1038/s41388-023-02602-z

**Published:** 2023-02-01

**Authors:** Cyrinne Achour, Devi Prasad Bhattarai, Paula Groza, Ángel-Carlos Román, Francesca Aguilo

**Affiliations:** 1 Department of Molecular Biology, Umeå University, SE-901 87 Umeå, Sweden; 2 Wallenberg Centre for Molecular Medicine, Umeå University, SE-901 87 Umeå, Sweden; 3 Department of Molecular Biology and Genetics, University of Extremadura, 06071 Badajoz, Spain

**Keywords:** RNA splicing, Breast cancer

## Abstract

Alternative splicing (AS) enables differential inclusion of exons from a given transcript, thereby contributing to the transcriptome and proteome diversity. Aberrant AS patterns play major roles in the development of different pathologies, including breast cancer. *N*^6^-methyladenosine (m^6^A), the most abundant internal modification of eukaryotic mRNA, influences tumor progression and metastasis of breast cancer, and it has been recently linked to AS regulation. Here, we identify a specific AS signature associated with breast tumorigenesis in vitro. We characterize for the first time the role of METTL3 in modulating breast cancer-associated AS programs, expanding the role of the m^6^A-methyltransferase in tumorigenesis. Specifically, we find that both m^6^A deposition in splice site boundaries and in splicing and transcription factor transcripts, such as *MYC*, direct AS switches of specific breast cancer-associated transcripts. Finally, we show that five of the AS events validated in vitro are associated with a poor overall survival rate for patients with breast cancer, suggesting the use of these AS events as a novel potential prognostic biomarker.

## Introduction

Alternative splicing (AS) of pre-mRNA is a crucial step in gene expression regulation that enables the coding diversity of the genome by selecting which transcript isoforms are expressed in a cell-specific and temporal manner [1]. AS results in the differential inclusion of exons that are joined by the spliceosome, a large multi-subunit complex comprised of five small nuclear ribonucleoprotein particles (snRNPs) and numerous proteins [2], yielding to multiple mRNA transcripts for the same given gene. AS is regulated by conserved *cis*-acting RNA elements responsible for the recruitment of splicing factors, which act either as enhancers or as silencers. The splicing outcome is determined by the composition of these RNA regulatory sequences, the differential G/C content between introns and exons, RNA secondary structures and exon/intron lengths [3]. In addition, AS is also influenced by chromatin conformation, histone modifications, DNA methylation, and the rate of transcription elongation [4].

AS functions in diverse biological processes including cell growth [5], stem cell-renewal and differentiation [6], and cell death [7], to name a few examples. Despite the advantage to expand cellular function, aberrant AS leads to human disease [8]. Indeed, recent advances in high-throughput technologies, which have enabled large-scale expression profiling of patient samples, have revealed widespread splicing alterations in both solid tumors and hematologic malignancies. Splice site mutations and/or dysregulated expression of splicing factors result in tumor-associated AS switches i.e. AS events in neoplastic tissues that are not detected in their normal counterparts. Tumor-associated AS switches have been linked to neoplastic transformation, tumor growth and progression, and resistance to therapy, and therefore can potentially be used as cancer biomarkers or as a tool for developing new-targeted cancer treatments [9].

Breast cancer is the most frequently diagnosed cancer and the leading cause of cancer-related mortality in women worldwide, being metastatic breast cancer incurable with the currently available therapies. Breast cancer is a heterogeneous disease classified into four molecular subtypes based on the presence of hormone receptors: luminal A (progesterone and estrogen receptor positive (PR+/ER+) and human epidermal receptor 2 negative (HER2-)), luminal B (PR+/ER+/HER2+), HER2 (PR-/ER-/HER2+), and triple-negative (PR-/ER-/HER2-). Treatment strategies differ according to the molecular subtype. Similar to other human tumors, breast cancer exhibits aberrant AS events due to mutations either within the splicing regulatory elements or at the splice sites of tumor suppressor genes, or dysregulated expression of the splicing machinery. Additionally, several studies have shown that *MYC* hyperactivation, a common feature in many human cancers, leads to transcriptional upregulation of splicing factors that direct breast cancer-associated AS switches promoting a malignant phenotype [10–12].

Similar to DNA and proteins, RNAs are also substrates for chemical modifications [13]. *N*^6^-methyladenosine (m^6^A), the most abundant internal modification in eukaryotic mRNA, has been shown to influence AS [14–17]. m^6^A is co-transcriptionally deposited by the methyltransferase-like 3 (METTL3) and METTL14 methyltransferase complex, which partially localizes to nuclear speckles, where splicing occurs [18–20]. It has been shown that depletion of the *Drosophila* METTL3 methyltransferase homolog, results in altered AS patterns that influence sex determination [21–23]. In addition, depletion of METTL3 led to an m^6^A-dependent RNA structural remodeling that alters the accessibility to m^6^A-binding proteins, affecting the recruitment of the splicing factor hnRNPC, and thereby influencing AS [24]. Indeed, hnRNPC has been recently reported to regulate AS in pancreatic ductal adenocarcinoma and non–small cell lung cancer [25, 26]. Another mechanism by which m^6^A regulates splicing is through the m^6^A reader YTHDC1 [14]. YTHDC1 binds to m^6^A-modified mRNA and recruits the splicing factor SRSF3, which promotes exon inclusion, but impedes the binding of SRSF10, which facilitates exon skipping. Moreover, increased m^6^A levels upon depletion of the eraser FTO promotes binding of SRSF2 resulting in exon inclusion in mouse preadipocytes [27]. However, an opposite trend was observed in a different cellular context. Specifically, in HEK293T cells another study showed that *FTO* knockout resulted in changes in splicing with exon skipping events being the most prevalent [28]. Although the function of m^6^A in AS has been questioned [29], it has been recently shown that deposition of m^6^A near splice junctions positively affects RNA splicing kinetics and modulates hnRNPG binding, an m^6^A reader which influences RNA polymerase II occupancy patterns and promotes exon inclusion [30, 31].

The last decade has unraveled multiple associations of m^6^A with different aspects of breast tumorigenesis [32]. However, it is still unclear whether this chemical mark contributes to tumor suppression or promotes oncogenicity. For instance, studies on METTL3 have revealed that it is overexpressed in breast cancer compared to normal mammary tissues, and its silencing in different breast cancer cell lines has been associated with increased apoptosis and decreased proliferation [33, 34]. On the contrary, another study reported that not only METTL3 but also other members of the writer complex such as METTL14 and WTAP are downregulated in breast cancer, suggesting that lower levels of m^6^A may contribute to breast tumorigenesis [35]. Similar contradictory findings are observed for other players of m^6^A modification, being writers, erasers or readers of m^6^A up- or down-regulated depending on the cellular context [32, 36]. Mechanistically, m^6^A may dictate the fate of tumor suppressor or oncogenic transcripts (e.g., *BCL2*, *BNIP3*, *c-MYC*, *CXCR4*, and *CYP1B1*), influence the treatment outcomes (e.g., resistance to tamoxifen or doxorubicin *via* methylation of AK4 or miRNA-221–3p) or regulate the stability of pluripotency factors (e.g., *Nanog* and *KLF4*), thus facilitating epithelial-mesenchymal transition (EMT), metastatic progression or the breast cancer stem cell phenotype, among others. Despite the plethora of information showing the implications of m^6^A in breast cancer, the biological relevance of m^6^A in breast tumor-associated AS switches is currently unexplored.

In this study, we identify an AS signature associated with the acquisition of the malignant phenotype of breast cancer in vitro. We describe that METTL3 regulates breast cancer-associated AS switches through a direct mechanism involving m^6^A deposition at the proximity of splice sites. Additionally, our data suggests indirect mechanisms by which METTL3 modulates AS in breast cancer through m^6^A deposition on splicing factors and transcriptional regulators of splicing factors such as *MYC*. Notably, our analyses reveal that m^6^A deposition correlates with intronic regions and depletion of METTL3 results in more exon inclusion for specific genes. Finally, we show that five of the in vitro validated AS events are associated with a worse prognosis in breast cancer patients, suggesting their use as potential prognostic biomarkers.

## Results

### Identification of AS events in non-tumorigenic and breast cancer cell lines

**Fig. 1:**
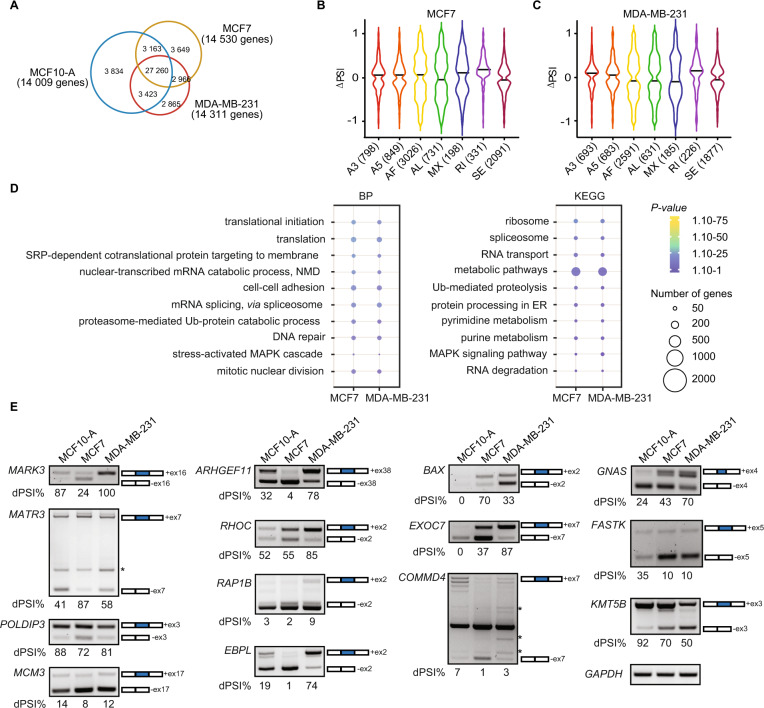
Identification of AS events in non-tumorigenic and breast cancer cell lines. **A** Venn diagrams showing the number of common alternative splicing events and genes in the non-tumorigenic mammary epithelial cell line MCF10-A and the breast cancer cell lines MCF7 and MDA-MB-231. Violin plots of changes of the significant percent splicing inclusion (∆PSI) in the breast cancer cell lines (**B**) MCF7 and (**C**) MDA-MB-231 related to the normal mammary epithelial cell line MCF10-A. **D** Dot plots representing the Gene Ontology enrichment (GO) analysis of the common spliced genes in MCF7 and MDA-MB-231. BP biological process, KEGG KEGG pathways. The size and the color of the dots are proportional to the number of genes enriched in each GO term and the significance of the enrichment (1.10^−75 ^< *P*-value < 1.10^−1^), respectively. **E** RT-PCR showing the different splicing events between the non-tumorigenic MCF10-A and breast cancer MCF7 and MDA-MB-231 cell lines. The number of the skipped exons are depicted for each transcript. The PSI was calculated in percentage for each gene. Non-specific bands are indicated with an asterisk.

To identify genome-wide differential splicing events (DSE) occurring during the acquisition of the breast cancer phenotype, we performed RNA-sequencing (RNA-seq) on a breast non-tumorigenic cell line (MCF10-A), and the commonly used luminal A (MCF7) and triple negative (MDA-MB-231) breast cancer models. Reads were then mapped to exon-splice junction sites to determine splicing events, including skipped exons (SE), retained introns (RI), mutually exclusive exons (MX), alternative first or last exons (AF or AL), and alternative 5´ or 3´splice sites (A5 or A3) (Supplementary Fig. 1A). First, we identified in total 37 680, 37 038 and 36 514 splicing events, corresponding to 14 009, 14 530 and 14 311 genes in MCF10-A, MCF7 and MDA-MB-231 cell lines, respectively (Fig. 1A). The differences of AS isoforms between the breast cancer cell lines and the non-tumorigenic MCF10-A cells were assessed by calculating the change in percent splicing inclusion (ΔPSI) and then with a false discovery rate (FDR), considering 0.05 as the threshold for a bona fide DSE (Supplementary Table S1 and Materials and Methods) [37]. In total, 8 024 and 6 886 corresponding to all different types of AS events (i.e. A3, A5, AF, AL, MX, RI and SE) were obtained in MCF7 and MDA-MB-231 in comparison to MCF10-A, respectively (Fig. 1B, C). Despite the majority of the splicing events being shared across the three cell lines (27 260 common events; Fig. 1A), the comparison revealed AS events that were unique to the breast cancer cell lines MCF7 and MDA-MB-231; AF and SE being the most represented categories (Fig. 1B, C). In addition, the ΔPSI values for both MCF7 and MDA-MB-231 had a uniform distribution between enhanced and repressed splice junctions (Supplementary Fig. 1B, C). We further performed Gene Ontology (GO) and Kyoto Encyclopedia of Genes and Genome (KEGG) pathway enrichment analysis for the DSE in MCF7 and MDA-MB-231. Only few genes (<50) were enriched in the GO biological process or KEGG pathway, including the terms “mRNA splicing, *via* spliceosome”, “cell-cell adhesion” and “MAPK signaling cascade”, amongst others (Fig. 1D). Additionally, the DSE in breast cancer cell lines were significantly enriched in the “nucleoplasm”, “cytosol”, “cytoplasm” and “nucleus” terms for the cellular components categories and enriched in the “protein binding and poly(A) RNA binding” category for the molecular function (Supplementary Fig. 1D).

We next validated selected AS from genes that were previously associated with different aspects of tumorigenesis (Fig. 1E and Supplementary Table 2). Upon validation of DSE, we observed that *MARK3* [38–40], *MATR3* [38–40], *POLDIP3* [41], and *MCM3* [39] displayed similar patterns between MCF10-A and MDA-MB-231 cell lines. For *MARK3* and *POLDIP3*, the skipping of exons 16 and 3 respectively, was more frequent in MCF7 compared to MCF10-A and MDA-MB-231, whilst the skipping of exon 7 for *MATR3* was less frequent in MCF7 cell line. MDA-MB-231 cells displayed distinct AS patterns for *ARHGEF11* exon 38 [10, 39, 40, 42], *RHOC* exon 2 [38, 39], *RAP1B* exon 2 [39], *EBPL* exon 2 [40]*, BAX* exon 2 [10, 38, 39], *EXOC7* exon 7 [43], and *COMMD4* exon 7 [39], whilst AS patterns for *GNAS* exon 4 [38, 39], *FASTK* exon 5 [41], and *KMT5B* exon 3 [39] were similar between MCF7 and MDA-MB-231 but differed from MCF10-A. There were transcript isoforms showing a positive exon inclusion index, which correlated to a significant negative skipping index of the same exon (e.g. *MARK3*, *RHOC* and *EBPL*). However, this was not the case for all the validated events (e.g. *EXOC7* and *RAP1B*), suggesting that multiple alternative exons can be spliced in a complex manner (Fig. 1E). Intriguingly, in the case of *BAX*, we were not able to amplify any isoform for the non-tumorigenic cell line, however we observed that the isoform including exon 2 was more expressed in MCF7 compared to MDA-MB-231, for which the expression of the isoform skipping exon 2 was higher.

Although the MCF10-A cell line is a widely used in vitro model as a surrogate for non-transformed mammary epithelial cells, the reliability for MCF10-A to mimic normal breast cells functions has been questioned [44]. Thus, we used the non-tumorigenic hTERT-HME1 cell line to confirm the DSE found in MCF10-A. Transcript isoforms expression was mostly similar between both cell lines, with the exception of *ARHGEF11* and *EXOC7*, where skipping of exons 38 and 7, respectively, were lower in hTERT-HME1. Additionally, *BAX* was more expressed in hTERT-HME1 compared to MCF10-A (Supplementary Fig. 1E). We next sought to extend the validation of the AS events to a panel of four breast cancer cell lines representative of different molecular subtypes of breast cancer i.e., T47D1 (luminal A), Hs578T (TNBC), MDA-MB-453 and SKBR3 (HER2). We observed that all of the analyzed transcripts displayed similar patterns between MCF7 and T47D1, both luminal A cell lines, with the exception of *RHOC* and *BAX* that were merely expressed in T47D1 cell line. Although both isoforms of *EXOC7* were expressed in T47D1, the long isoform including exon 7 was more abundant in T47D1 compared to MCF7 (Supplementary Fig. 1E). MDA-MB-231 and Hs578T, both representing the TNBC subtype, presented a similar splicing pattern with the exception of *RHOC*, where skipping of exon 2 was more prevalent in Hs578T than in MDA-MB-231 (Supplementary Fig. 1E). Strikingly, both HER2+ cell lines analyzed presented opposite splicing patterns for *ARHGEF11*, *BAX*, and *EXOC7* (Supplementary Fig. 1E). Altogether, our data show that the AS signature we observed initially can be extended to other breast cancer cell lines although with intrinsic variability within the same breast cancer subtype.

### METTL3 enhances breast cancer growth

**Fig. 2:**
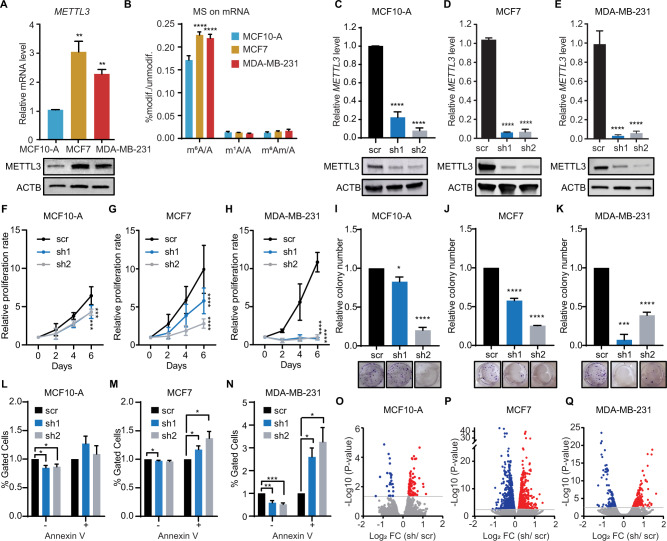
METTL3 promotes cell growth in breast cancer cell lines. **A** RT-qPCR analysis of *METTL3* mRNA level (upper) in the non-tumorigenic mammary epithelial cell line MCF10-A and the breast cancer cell lines MCF7 and MDA-MB-231. *METTL3* is normalized to *β-ACTIN*. Western blot of METTL3 (lower) on whole cell extracts (WCE) from MCF10-A, MCF7 and MDA-MB-231 cell lines. β-ACTIN (ACTB) is used as the loading control. **B** LC-MS/MS quantification of m^6^A, m^1^A and m^6^A_m_ in mRNA of MCF10-A, MCF7 and MDA-MB-231 cell lines. Methylated adenosines are normalized to the total of unmodified adenosines. RT-qPCR analysis and western blot of METTL3 mRNA and protein levels, respectively in *METTL3* knockdown (sh1 and sh2) and scramble (scr) control in MCF10-A (**C**), MCF7 (**D**) and MDA-MB-231 (**E**) cell lines. METTL3 is normalized to β-ACTIN. Cell proliferation rate of scramble (scr) and *METTL3* knockdowns (sh1 and sh2) in MCF10-A (**F**), MCF7 (**G**) and MDA-MB-231 (**H**) assessed over 4 days. Colony formation assay in MCF10-A (**I**), MCF7 (**J**) and MDA-MB-231 (**K**) cells in scramble (scr) and upon depletion of METTL3 (sh1 and sh2) at 7 days after seeding. Quantification of the relative number of colonies was calculated with scr set as 1. Percentage of apoptotic cells in control and METTL3 depleted cells in MCF10-A (**L**), MCF7 (**M**), and MDA-MB-231 (**N**) cell lines. + indicates Annexin V positive cells; and – indicates Annexin V negative cells. Volcano plots representing the Log_2_ fold change of differentially expressed genes upon *METTL3* knockdown (sh1 and sh2) in MCF10-A (**O**), MCF7 (**P**) and MDA-MB-231 (**Q**) cell lines in comparison to control cells (scr). The significant up- and down-regulated genes are shown in red and blue, respectively. *P*-value < 0.05. Data are mean ± SEM; *n* = 3; ****p < 0.0001; ***p < 0.001; **p < 0.01; *p < 0.05.

Deposition of m^6^A, catalyzed by METTL3, modulates nearly every aspect of the mRNA lifecycle, including AS [19, 45]. To determine whether m^6^A regulates AS in breast cancer, we first assessed the expression of METTL3 across non-tumorigenic and breast cancer cell lines. MCF7 and MDA-MB-231 exhibited increased METTL3 expression compared to MCF10-A (Fig. 2A), which was also observed in the broader panel of breast cancer cell lines with the exception of the Tamoxifen resistant cell line T47D1 (Supplementary Fig. 2B). METTL14, required for the catalytic activity of METTL3, was also upregulated in the breast cancer cell lines (Supplementary Fig. 2A, C). We then sought to analyze the expression level of other m^6^A regulators across the three cell lines. The writer *VIRMA* was upregulated in both breast cancer cell lines, whereas *HAKAI* was only significantly upregulated in MCF7 (Supplementary Fig. 2D, E). Strikingly, *WTAP* and the eraser *FTO* were downregulated in breast cancer cell lines compared to normal epithelial cells, whilst no changes were observed for *ALKBH5* (Supplementary Fig. 2F–H). Furthermore, the expression of METTL5, which has been reported to promote breast cancer growth [46], was lower in MCF7 and MDA-MB-231 compared to MCF10-A at the mRNA level, but higher at the protein level. This suggested that post-transcriptional regulation of METTL5 may occur during the acquisition of the breast cancer phenotype (Supplementary Fig. 2A, I). Even though METTL5 is overexpressed in the breast cancer cell lines, it is known to deposit m^6^A in the 18S ribosomal RNA; and given that m^6^A levels in mRNA were also higher in MCF7 and MDA-MB-231 cells (Fig. 2B), we aimed to understand the contribution of m^6^A in mRNA during breast tumorigenesis. To this end, we depleted METTL3 in MCF10-A, MCF7 and MDA-MB-231 cells using two distinct short-hairpin RNAs (shRNAs) targeting *METTL3* (thereafter referred to as sh1 and sh2) to ensure that the observed phenotype is not due to shRNA off-target effects (Fig. 2C–E). Consistently, m^6^A levels on mRNA were significantly lower upon silencing of *METTL3* compared to control cells (Supplementary Fig. 2J–L). *METTL3* knockdown cells exhibited a significant defect in proliferation, which was accentuated in the breast cancer cell lines MCF7 and MDA-MB-231 compared to *METTL3* knockdowns in MCF10-A (Fig. 2F–H). Similarly, silencing of *METTL3* in MCF7 and MDA-MB-231 led to a reduced number of colonies formed whereas MCF10-A cells depleted of METTL3 displayed less defects (Fig. 2I–K). Additionally, apoptotic rate was increased upon *METTL3* knockdown (Fig. 2L–N). Taken together, these results suggest that METTL3 promotes breast cancer growth.

To gain further insight into the molecular mechanism by which METTL3 promotes cell growth in breast cancer, we next performed RNA-seq upon silencing of *METTL3* in MCF10-A, MCF7 and MDA-MB-231 cells. Although silencing of *METTL3* did not dramatically affect the steady state mRNA levels, differences in gene expression (DEG) were more exacerbated in the breast cancer cell lines compared to MCF10-A cells (Fig. 2O–Q; Supplementary Fig. 2M; Supplementary Table S3). GO analysis for biological processes of up-regulated genes upon silencing of *METTL3* in MCF7 was enriched for response to drug, cell-cell adhesion, and sulfation (Supplementary Fig. 2N). Sulfation plays an important role in the anti-tumorigenic mechanism of tamoxifen, suggesting that METTL3 could increase the sensibility of hormone therapy in the luminal A breast cancer subtype. GO analysis of down-regulated genes revealed generic functions, which included response to hypoxia and drug, inactivation of MAPK activity, and regulation of apoptotic processes (Supplementary Fig. 2O). In MDA-MB-231 depleted of METTL3, GO analysis of up-regulated genes revealed generic functions (Supplementary Fig. 2P), which included the tumor suppressors *RARRES2*, *ITLN1*, *CALCR* and *UTRN*. On the other hand, GO analysis of down-regulated genes showed that cellular response to lipopolysaccharide, angiogenesis, negative regulation of cell proliferation, and cell adhesion were among the most enriched biological processes (Supplementary Fig. 2Q).

### METTL3 regulates AS in breast cancer cell lines

**Fig. 3:**
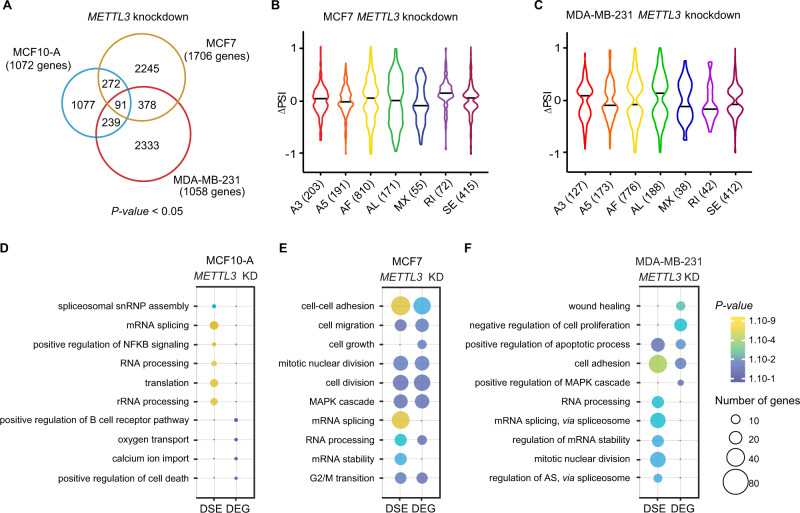
METTL3 modulates AS in breast cancer cell lines. **A** Venn diagrams showing the common alternative splicing events between knockdowns of *METTL3* in MCF10-A (blue), MCF7 (yellow) and MDA-MB-231 (red). The corresponding total number of genes is indicated in brackets. *P*-value < 0.05, statistically significant. **B**, **C** Violin plots of the significant percent splicing inclusion (∆PSI) in knockdowns of *METTL3* in MCF7 and MDA-MB-231 cells related to control cells. Dot plots of the GO enrichment analysis of the differentially expressed (DEG) and spliced (DSG) genes in (**D**) MCF10-A, (**E**) MCF7 and (**F**) MDA-MB-231 upon depletion of METTL3. The size and the color of the dots are proportional to the number of genes enriched in each GO term and to the significance of the enrichment (1.10^−9^ < *P*-value < 1.10^−1^), respectively.

We next sought to assess how METTL3 influences AS in breast cancer cells. As explained above, reads were mapped to exon-splice junction sites to identify DSE, and both datasets, from sh1 and sh2, were combined to obtain the most significant AS events (Supplementary Fig. 3A, B; Supplementary Table 4). Upon silencing of *METTL3*, we identified 1 679 DSE (1 072 genes), 2 986 DSE (1 706 genes) and 3 041 DSE (1 058 genes) in MCF10-A, MCF7 and MDA-MB-231, respectively [FDR] < 0.05; Fig. 3A). Thus, METTL3 depletion accompanied broader modulations in the AS landscape of breast cancer cells lines compared to the non-tumorigenic MCF10-A, suggesting a critical role of METTL3 in regulating tumor-associated AS switches (Supplementary Fig. 3C). Noteworthy, alterations in DSE upon *METTL3* knockdown were not due to transcriptional changes as gene expression levels were not correlated to ΔPSI (Supplementary Fig. 3D, E). GO analysis revealed that the common METTL3-regulated AS events in breast cancer cell lines were enriched for the terms “translation”, “regulation of apoptotic process” and “regulation of growth”, suggesting that METTL3 may affect breast tumorigenesis through AS regulation (Supplementary Fig. 3F, G). Strikingly, GO categories related to “splicing” and “alternative splicing” were highly represented. All types of AS events were affected upon knockdown of *METTL3*, most of the events corresponding to AF in both MCF7 (810 DSE) and MDA-MB-231 (776 DSE) (Fig. 3B, C).

To assess the functional impact of METTL3 in breast cancer, we next performed GO analysis of the DEG and the DSE of *METTL3* knockdown in MCF10-A, MCF7 and MDA-MB-231 cell lines (Fig. 3D–F). GO analysis of the DEG or the DSE in MCF10-A depleted of METTL3 did not show terms enriched in categories related to tumorigenesis, and only few DSE were associated to splicing or translation, and these genes were not differential expressed (Fig. 3D). However, in breast cancer cell lines, biological processes frequently altered during tumor progression and metastasis were amongst the most enriched terms. In particular, we observed a common significant enrichment in GO terms associated with “cell adhesion” in both cell lines, and “MAPK cascade” and “apoptosis” in MCF7 and MDA-MB-231, respectively (Fig. 3E, F). Additionally, we found that the terms “mRNA stability”, “mRNA splicing” and “RNA processing” were specifically enriched in both MCF7 and MDA-MB-231 for the DSE, but these terms were not found among genes whose mRNA levels were affected by *METTL3* silencing. Overall, this data supports the idea that m^6^A may regulate breast tumorigenesis by influencing multiple pathways, including AS of splicing factors and other RNA-binding proteins.

### Intronic m^6^A modification affects AS

**Fig. 4:**
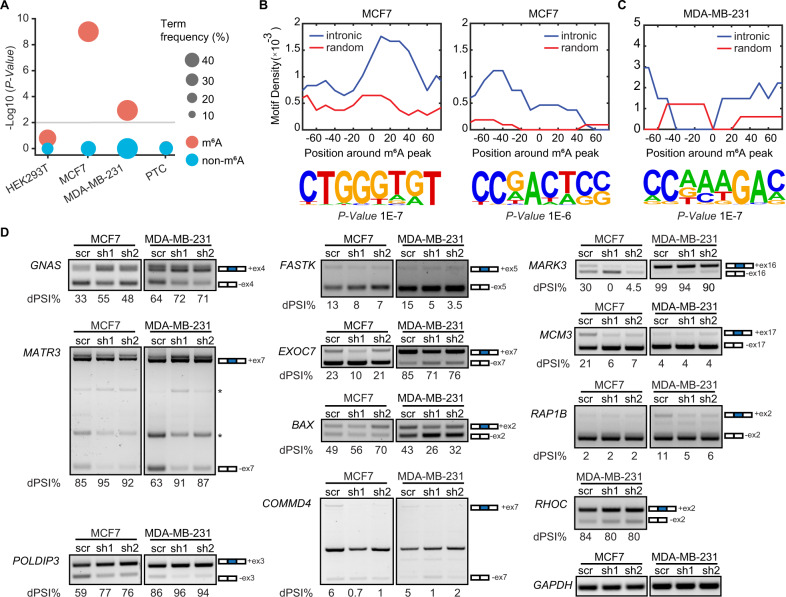
METTL3 influences AS *via* m^6^A deposition. **A** Dot plot representing the level of statistical significance of AS transcripts harboring m^6^A (red) or non-m^6^A modified AS transcripts (blue), in chromatin-bound transcripts dataset from HEK293T, MCF7, MDA-MB-231, and transcripts with premature termination codons (PTC) dataset from human glioblastoma [48]. The size of the dots is proportional to the frequency of the events. *P*-value < 0.01, statistically significant. Motif density of m^6^A peaks in the –80 to +80 nt region around the m^6^A peak in intronic or random regions (upper panels) and the corresponding HOMER motifs outputs (lower panels) in (**B**) MCF7 and in (**C**) MDA-MB-231. **D** RT-PCR of AS genes in *METTL3* knockdown in breast cancer cell lines MCF7 and in MDA-MB-231 cells. The number of the skipped exons are depicted for each transcript. The PSI was calculated in percentage for each gene. Non-specific bands are indicated with an asterisk.

To identify whether METTL3 modulates AS through m^6^A deposition at the proximity of splice sites, we analyzed available m^6^A-RNA immunoprecipitation sequencing (MeRIP-seq) data from chromatin-associated RNAs in HEK293T cells. Although some of the transcripts that underwent DSE upon *METTL3* knockdown harbored the m^6^A mark at exon-intron junction sequences (e.g. *DLG5*, *LARGE1*, *INO80C*), we could not detect any significant correlation between intronic m^6^A deposition and AS (Fig. 4A). Using previously published available MeRIP-seq datasets, we next examined the distribution of m^6^A sites in introns flancking SE retrieved in our data in MCF7 and MDA-MB-231 cell lines [47]. We found a significant enrichment between m^6^A deposition at exon-intron junction boundaries and processing efficiency (Fig. 4A; Supplementary Fig. 4A, B). Notably, more than half of the introns flancking the differentially SE that we identified in MCF7 and MDA-MB-231 harbor m^6^A (Supplementary Fig. 4C). Sequence logo analysis revealed the presence of highly enriched non-DRACH (D = A, G, U; R = A, G; H = A, C, U) motifs in the regions ±150 nt around the m^6^A peak summit compared to randomly generated 300 nt intervals (Fig. 4B, C and Supplementary Fig. 4D). Altogether, these results suggest that m^6^A could directly regulate AS in our cellular models, and that the DSE are cell-type specific. Moreover, these isoforms had coding potential as they were not enriched for PTCs, stop codons that occur >50 nucleotides upstream of the splice junction [48], which would result in nonsense-mediated decay (Fig. 4A). We next performed GO analysis of the m^6^A datasets for MCF7 and MDA-MB-231 and observed that again “mRNA and RNA splicing” were amongst the most enriched terms (Supplementary Fig. 4E). Similar to METTL3-dependent AS switches (Fig. 3E, F), m^6^A deposition was also prominent in categories important for breast cancer progression and metastasis.

We then sought to identify whether intronic m^6^A deposition is associated with AS in our breast cancer models. To do so, we performed RT-PCR of nine transcripts harboring intronic m^6^A and two transcripts lacking m^6^A, in MCF7 and MDA-MB-231 upon silencing of *METTL3* (Fig. 4D and Supplementary Fig. 4F). We found that METTL3 depletion promoted exon inclusion of the alternative exons of *GNAS*, *MATR3*, *POLDIP3* and promoted skipping of the alternative exons of *COMMD4, MARK3* and the non-m^6^A modified transcripts *FASTK* and *EXOC7* in both breast cancer cell lines. Depletion of METTL3 led to a decrease of the inclusive isoform of *MCM3* in MCF7 whereas no significant difference was observed between the knockdowns and control cells in MDA-MB-231. Additionally, less exon skipping was observed for *BAX* upon depletion of METTL3 in MCF7 in opposite to MDA-MB-231. In contrast, a decrease of the inclusive isoform was observed for *RAP1B* upon knockdown of *METTL3* in MDA-MB-231 in comparison to MCF7, for which there was no change after depletion of METTL3. Altogether, these results indicate that intronic m^6^A deposition is associated with AS influencing the acquisition of breast cancer phenotype characteristics.

### METTL3 indirectly influences AS through MYC regulation

The expression of splicing factors is generally dysregulated in breast cancer leading to tumor-specific AS events [49–51]. Thus, we analyzed the expression of the spliceosome-associated proteins in the SF3A/B, the U2AF core complex, the hnRNP family and key splicing regulators, in control and knockdown of *METTL3* in all our cellular models (Supplementary Fig. 5A). In the SF3A/B sub-complex, *SF3A3* expression was higher in MDA-MB-231 in agreement with recent observations stating that SF3A3 predicts molecular and phenotypic features of aggressive human breast cancers [11]. Likewise, *SF3B4* displayed higher expression levels not only in MDA-MB-231 but also in MCF7 cells. The transcript levels of SR proteins and hnRNPs during breast tumorigenesis were heterogeneous, with some components being up-regulated (e.g. *SRSF1*, *SRSF3*, *SRSF9*, *TRA2B, ILF2, ILF3, PTBP1*, and *hnRNPA2B1*) and others down-regulated (e.g. *SRSF5* and *SRSF8*) in MCF7 and MDA-MB-231 compared to MCF10-A. Noteworthy, hnRNPC and hnRNPA2B1 have been identified as m^6^A RNA readers [17, 52], and although the latter is m^6^A modified, no effect on *hnRNPA2B1* mRNA levels were observed upon *METTL3* knockdown. Likewise, we found that several other splicing factors of the aforementioned complexes were targets of m^6^A modification, although we did not detect a major effect on METTL3-mediated expression regulation by assessing their RNA steady levels (Supplementary Fig. 5A). This is consistent with GO analysis of our RNA-seq data that did not reveal dysregulation of splicing-associated categories (Supplementary Fig. 2M–P).

**Fig. 5:**
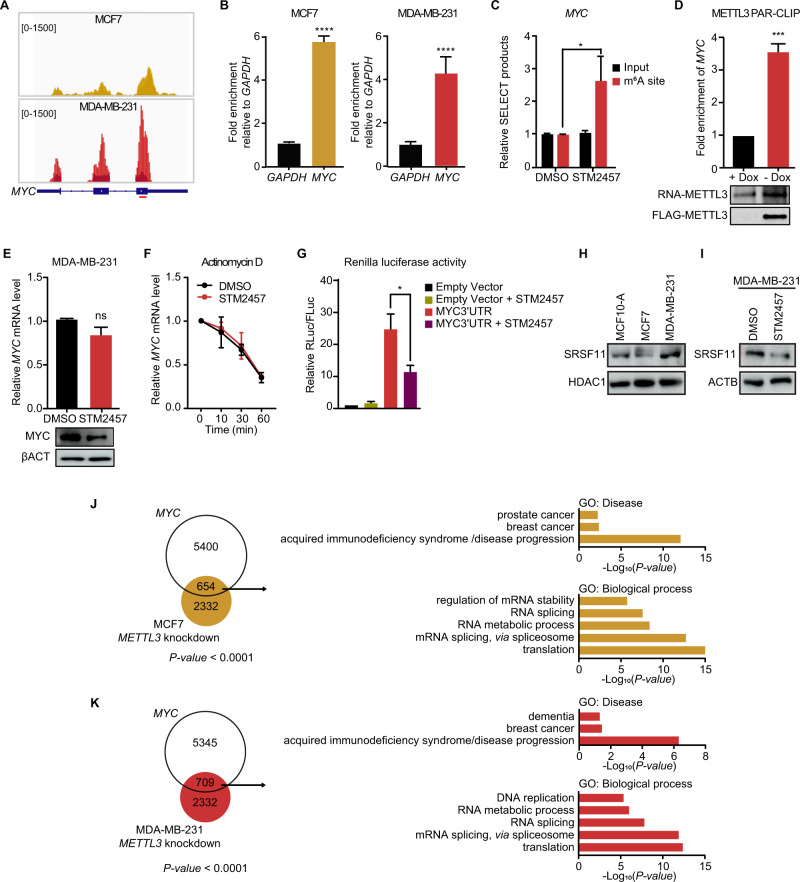
m^6^A motifs in *MYC* 3´UTR promotes the translation of *MYC* mRNA. **A** m^6^A peak distribution in *MYC* mRNA in MCF7 (left panel) and MDA-MB-231 (right panel) visualized in IGV. Input reads are represented in darker colors and the enriched RNA immunoprecipitated in yellow (MCF7) or red (MDA-MB-231). The amplified region by qPCR is depicted with a red line below *MYC* gene body. **B** RT-qPCR of m^6^A RNA immunoprecipitation (MeRIP) showing the enrichment of m^6^A in *MYC* relative to *GAPDH* in MCF7 (left) and MDA-MB-231 (right). **C** Relative level of SELECT products specific to m^6^A site in *MYC* 3´UTR, using total RNA from DMSO treated or STM2457 treated MDA-MB-231 cells. **D** RT-qPCR analysis of *MYC* after FLAG-METTL3 immunoprecipitation performed in control cells (+Dox) or in cells overexpressing Tet-off FLAG-METTL3 (-Dox) in MDA-MB-231 cells. **E** RT-qPCR analysis of *MYC* mRNA (upper panel) and western blot for MYC (lower panel) in MDA-MB-231 upon STM2457 treatment. βACTIN is used as loading control. **F** RT-qPCR analysis of *MYC* mRNA after treatment with actinomycin D at the time points 0, 10, 30 and 60 min in MDA-MB-231 control and treated with STM2457. **G** Relative Renilla luciferase activity of the psiCHECK2-*MYC 3´UTR* in MDA-MB-231 cells treated with DMSO (control) or with STM2457 for 48 h. Control cells were transfected with psiCHECK2 empty vector. Renilla luciferase activity was measured and normalized to Firefly luciferase. Data are mean ± SEM; *n* = 3 or 4; ****p < 0.0001; ***p < 0.001; *p < 0.05. In **A**, **D**, **E**, and **G** *P*-values were determined by two-tailed *t*-test; in **C** *P*-values were determined by one-tailed *t*-test. **H** Western blot showing the overexpression of SRSF11 in MDA-MB-231 in comparison to MCF10-A and MCF7. HDAC1 is used as loading control. **I** Western blot assessing the expression of SRSF11 in MDA-MB-231 upon STM2457 treatment. βACTIN is used as loading control. **J** Overlaps between AS events of knockdown of *METTL3* in MCF7 and MYC-associated AS events (left panel); *P*-value < 0.0001. GO analysis of the common genes between AS events between knockdown of *METTL3* in MCF7 and MYC-associated AS events (right panel); *P*-value < 0.05. **K** Overlaps between AS events of knockdown of *METTL3* in MDA-MB-231 and MYC-associated AS events (left panel); *P*-value < 0.0001. GO analysis of the common genes between AS events in knockdown of *METTL3* in MDA-MB-231 and MYC-associated AS events (right panel); *P*-value < 0.05.

Overexpression or hyperactivation of the transcription factor MYC occurs in most human cancers, and previous studies have illustrated that *MYC* mRNA harbors m^6^A [53, 54]. Indeed, this was the case in both MCF7 and MDA-MB-231 breast cancer cell lines (Fig. 5A, B). We validated the presence of m^6^A at one specific site located in the last exon of *MYC*, where it is highly enriched, by an antibody-independent method, namely SELECT (single‐base elongation‐ and ligation‐based qPCR amplification) [55]. To this end, MDA-MB-231 cells were incubated with the selective METTL3 inhibitor STM2457 (STORM Therapeutics). The SELECT assay relies on the fact that m^6^A impairs the cDNA production, thus, we observed an increase in the efficiency of the qPCR amplification when the m^6^A mark was depleted (Fig. 5C). Binding of METTL3 to *MYC* mRNA was further validated by photoactivatable ribonucleoside-enhanced crosslinking and immunoprecipitation (PAR-CLIP) in an MDA-MB-231 cell line with doxycycline-mediated silencing of *METTL3* (Fig. 5D). Western blotting revealed that inhibition of METTL3 by STM2457 led to reduced levels of MYC protein although *MYC* mRNA stability was unchanged compared to control cells (Fig. 5E, F). To assess whether MYC expression is mediated by m^6^A deposition, we transfected MDA-MB-231 cells with a luciferase reporter that contains the wild type sequence of *MYC* 3´UTR downstream of *Renilla* in which the consensus m^6^A motifs were ablated. The consensus m^6^A sites in *Firefly luciferase* were also ablated. Renilla activity was decreased upon treatment with the METTL3 inhibitor, indicating that m^6^A in *MYC* 3´UTR is sufficient to regulate MYC expression (Fig. 5G).

MYC-mediated upregulation of core splicing factors is critical for sustaining growth in MYC-driven tumors [12, 56]. Hence, given that translation of SRSF11 is enhanced under MYC hyperactivation [11] and overexpressed in MDA-MB-231 (Fig. 5H), we further evaluated SRSF11 expression upon treatment with STM2457 (Fig. 5I). Following the same pattern as MYC, the protein level of SRSF11 was reduced upon inhibition of METTL3, suggesting that METTL3 modulates AS by mediating the expression of a subset of spliceosomal components through MYC. Next, we overlapped the DSE datasets from knockdown of *METTL3* in MDA-MB-231 and MCF7 with DSE from MYC-driven AS switches generated from public RNA-seq datasets. We found that ~22% and ~30% of the METTL3-associated AS events in MCF7 and MDA-MB-231, respectively, overlapped with the MYC-associated AS events (Fig. 5J–K). GO analysis of the common AS events in the Disease category revealed an enrichment for the term “breast cancer” (Fig. 5J–K). Additionally, GO for the biological processes category were enriched for terms related to splicing such as “mRNA splicing, *via* spliceosome”, “RNA splicing”, and “mRNA processing”, as well as terms related to “autophagy”, “cell division”, and “regulation of cell cycle“. Overall, these results suggest that METTL3 may indirectly regulate AS in breast cancer *via* m^6^A deposition in *MYC* mRNA.

### Identification of breast cancer prognosis-related AS events

**Fig. 6:**
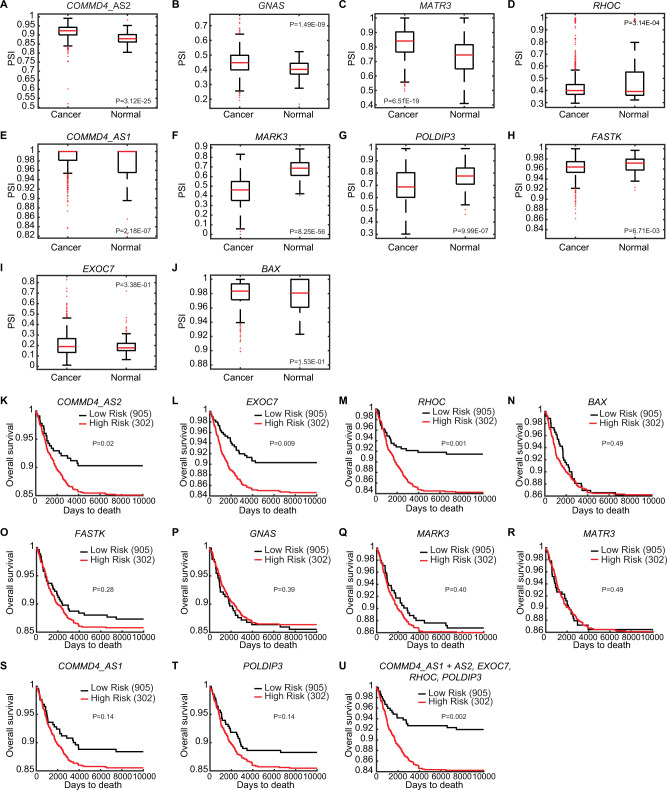
Identification of breast cancer prognosis-related AS events. PSI values were analyzed in breast cancer patients (1094 samples) and normal samples (113 samples) for the AS events tested in (**A**) *COMMD4*_AS2, (**B**) *GNAS*, (**C**) *MATR3*, (**D**) *RHOC*, (**E**) *COMMD4*_AS1, (**F**) *MARK3*, (**G**) *POLDIP3*, (**H**) *FASTK*, (**I**) *EXOC7*, (**J**) *BAX*. Data were taken from the TCGA SpliceSeq database. Kaplan–Meier plots of overall survival (OS) for breast cancer patients classified according to the AS events expression (low or high) for (**K**) *COMMD4_AS2*, (**L**) *EXOC7*, (**M**) *RHOC* (**N**) *BAX*, (**O**) *FASTK*, (**P**) *GNAS*, (**Q**) *MARK3*, (**R**) *MATR3*, (**S**) *COMMD4*_AS1, (**T**) *POLDIP3*. **U** OS rate for the combination of *COMMD4_AS1*, *COMMD4_AS2*, *EXOC7*, *RHOC* and *PODLIP3*. *p* < 0.05, statistically significant.

To interrogate whether the DSE events that were validated in the MCF7 and MDA-MB-231 cell lines could define a breast cancer-associated AS signature in patients, we analyzed The Cancer Genome Atlas (TCGA) SpliceSeq datasets as well as their associated clinical information for *COMMD4*, *GNAS, MATR3*, *RHOC*, *MARK3*, *POLDIP3, FASTK, BAX*, and *EXOC7*. For *COMMD4*, two alternative isoforms were analyzed (namely *COMMD4_*AS1 and *COMMD4_*AS2). We observed that the AS events for *COMMD4_*AS2, *GNAS, MATR3,* *COMMD4*_AS1 and *RHOC* displayed a significant higher PSI value in cancer patients than in normal samples (Fig. 6A–E), while the PSI values were significantly lower for *MARK3*, *POLDIP3* and *FASTK* in patients with breast cancer (Fig. 6F–H). However, our analysis showed no difference in the PSI values for *BAX*, and *EXOC7* between the cancer patients and normal samples (Fig. 6I, J). We next employed the same tool to interrogate the PSI values for each DSE mentioned above at different grades of breast cancer (Supplementary Fig. 6A–J). In comparison to the normal samples (M0), AS switches occurring in patients with breast cancer metastasis (M1) were significantly different in *COMMD4_*AS2, *MARK3*, and *MATR3* (Supplementary Fig. 6A–C). Inclusions of alternative exons were more prevalent for *COMMD4_*AS2 and *MATR3*, while exclusions were more prevalent for *MARK3. POLDIP3*, *FASTK*, *COMMD4*_AS1, *GNAS* and *RHOC* did not show a significant difference between patients with metastasis and normal samples (Supplementary Fig. 6D–H). However, exclusions of alternative exons were more prevalent for *POLDIP3*, *FASTK* and *COMMD4*_AS1 in patients with no metastasis (M0) compared to normal samples, whereas inclusions were more prevalent for *GNAS* and *RHOC*. Additionally, no significant difference in the PSI values were found for *BAX* and *EXOC7* in patients compared to the normal samples (Supplementary Fig. 6I, J).

We next investigated more in depth the AS events within the different stages of breast cancer i.e., from stage I, where the tumor has not spread to lymph nodes or outside the breast, to stage IV in which the cancer has spread to distant organs. Although we found variabilities along the stages, which reflects the heterogeneity of this disease, *COMMD4_*AS2, *MARK3*, *MATR3*, *POLDIP3*, *COMMD4_*AS1, and *GNAS* underwent AS switches in almost all stages of breast cancer, while *RHOC* displayed significant AS switches during stage IIB (Supplementary Fig. 6K–Q). Nonetheless, *FASTK*, *BAX* and *EXOC7* did not show a significant difference between patients with breast cancer at different stages and the normal samples (Supplementary Fig. 6R–T).

To further elucidate the link between METTL3 and the breast cancer-associated AS signature in patients, we analyzed the correlation between *METTL3* expression and the aforementioned DSE events using TCGA datasets. We found that AS switches of *COMMD4*_AS1, *COMMD4*_AS2, *GNAS*, *MARK3*, *MATR3*, *FASTK*, and *EXOC7* correlated with METTL3 expression (*p* < 0.05) (Supplementary Fig. 7A–G). Additionally, *COMMD4*_AS1, *GNAS*, *POLDIP3, FASTK*, and *RHOC* AS were significantly associated with *METTL3* deletion (Supplementary Fig. 7H–L) whereas AS of *EXOC7* correlated with both deletion and gain of *METTL3* in invasive breast carcinoma (Supplementary Fig. 7M).

We then explored the relationship between DSE and the prognosis of breast cancer patients. To this end, the overall survival rate of breast cancer patients was divided in high or low risk groups in association to each AS event. Kaplan–Meier curves for *COMMD4_*AS2, *EXOC7* and *RHOC* (*P-value* < 0.05) revealed that patients with a high-risk score had a worse prognosis (Fig. 6K–M), while the AS events for *BAX*, *FASTK*, *GNAS*, *MARK3*, and *MATR3* were not associated to the survival rate (Fig. 6N–R). Additionally, *COMMD4_AS1* and *POLDIP3* (Fig. 6S–T) presented a trend towards a worse prognosis.

Taken together, the AS events analyzed in *COMMD4_AS2*, *EXOC7*, *RHOC*, *COMMD4_*AS1 and *POLDIP3* were related to a worse breast cancer prognosis (Fig. 6U), and could constitute potential prognosis biomarkers. Furthermore, *COMMD4*, *MARK3*, *MATR3*, *POLDIP3* could be used as biomarkers to specify the stage of the disease.

## Discussion

In the last decade, m^6^A has been established as an important layer of post-transcriptional control of gene expression, and its dysregulated deposition has been defined to be critical for breast cancer initiation, progression and metastasis [32]. Although several studies have shown the role of m^6^A in splicing regulation [8, 14, 27, 30, 45], to our knowledge, the function of m^6^A in breast cancer-associated AS switches has not been reported yet. Here, we identify genome-wide METTL3-regulated AS events in breast cancer cell lines, and reveal both direct and indirect connections between m^6^A and AS.

We profiled the transcriptome of the normal epithelial MCF10-A, MCF7 and MDA-MB-231 cell lines, with the last two representing distinct breast cancer subtypes. We observed global changes in AS of common transcripts across the three cell lines. Analysis of the AS landscape also revealed a cell-type specific AS signature of a number of genes involved in critical functions for breast tumorigenesis, such as mitotic nuclear division, MAPK signaling cascade, and DNA repair. We validated a selection of AS events, many of them with a known function in migration, invasion and EMT not only in MCF7 and MDA-MB-231 but also in a broader panel of cell lines representing the distinct molecular subtypes of breast cancer. Interestingly, we found some of these splicing patterns to be similar between the non-tumorigenic MCF10-A and the invasive MDA-MB-231 cell lines. MCF10-A can be grown three-dimensionally (3D) in matrigel mimicking the acinar structure of the mammary gland [57], and opposite splicing patterns between 3D and 2D MCF10-A cultures have been reported [58]. Hence, future studies should address which cellular conditions, 3D or 2D, are more faithful to non-tumorigenic epithelial cells. Additionally, a molecular characteristic of MCF10-A includes amplification of *MYC*, which has been reported to play a critical role in oncogenic AS switches [12, 56]. Nevertheless, the majority of transcript isoform expressions from MCF10-A were similar to the AS found in the normal mammary hTERT-HME1 cell line, and the majority of the validated AS events displayed cell-type specific but also common patterns between both breast cancer cell lines.

Although METTL3 has been extensively studied for over a decade, our knowledge about its role in cancer is still limited. Thus, we performed loss-of-function experiments to assess the function of METTL3 in breast tumorigenesis. Depletion of METTL3 reduced m^6^A levels, and resulted in proliferation defects and increased apoptosis, suggesting that METTL3 functions as an oncogene in breast cancer. Importantly, such proliferation defects were more accentuated in MCF7 and MDA-MB-231 compared to normal mammary epithelial cells. Additionally, our RNA-seq data showed that genes involved in proliferation and migration, including cell-cell adhesion, were altered upon depletion of METTL3 in breast cancer cell lines. Remarkably, no major changes in RNA steady state levels were observed in MCF10-A after depletion of METTL3. We also interrogated genome-wide METTL3-regulated AS events in breast cancer cell lines. Our data further demonstrated that METTL3 regulates tumor-associated AS switches in breast cancer, and that METTL3 depletion causes mainly alternative first and exon skipping events. It has been shown that the reader YTHDC1 binds to m^6^A sites and recruits the splicing factor SRSF3 to promote exon inclusion [14]. Hence, it is plausible that lower m^6^A deposition upon depletion of METTL3 leads to decreased YTHDC1 binding, which in turn promotes exon skipping. Differentially expressed and spliced genes in *METTL3* knockdown MCF7 cells were enriched in cancer-associated categories such as MAPK cascade, cell migration and cell-cell adhesion. However, in MDA-MB-231 we found that most of the biological processes were subjected exclusively to regulation by AS but not by changes in gene expression or vice versa. For instance, there was a striking enrichment for splicing-related categories in DSE. Hence, it is likely that the observed METTL3-mediated AS phenotype also results from differential splicing events occurring in transcripts encoding splicing factors, although this hypothesis warrants further study. Nevertheless, our findings highlight that many biological processes occurring in breast cancer cells are regulated only by METTL3-induced AS, expanding the repertoire of functions of METTL3 in tumorigenesis.

Our findings furthermore revealed a significant m^6^A deposition near splice junction sites of mRNAs. This was only true when comparing genome-wide DSE with MeRIP-seq data from the same cell line, emphasizing the notion of a cell-type specific AS and m^6^A signature. Interestingly, intronic m^6^A deposition associated to DSE was mostly found in non-DRACH sequences. One limitation of our study is that we used publicly available MeRIP-seq data by which m^6^A regions are detected as the enrichment of immunoprecipitated RNA relative to input RNA. Therefore, the m^6^A site at nucleotide resolution and the m^6^A stoichiometry cannot be interrogated with this conventional antibody-based approach, thereby hampering the identification of direct m^6^A effects on AS. Noteworthy, new technologies such as DART-seq and m^6^A-SAC-seq have identified more m^6^A sites than previously known, yet this data is not available for the cell lines used in our study [59, 60]. This suggests that the plethora of potential splice sites regulated by m^6^A is underestimated. Additionally, single cell analysis has uncovered substantial heterogeneity of m^6^A sites across individual cells [61]. Indeed, many m^6^A sites that are highly methylated at the population level show a low or an absence of methylation in a substantial number of individual cells, which could further impede the assessment of m^6^A-associated DSE. Noteworthy, gene transcripts arising after depletion of METTL3 likely encoded for functional proteins, as we did not observe an enrichment for PTC. Thus, the METTL3-dependent AS switch may generate new isoforms (e.g. *MATR3*) or alter the proportion of existing isoforms (e.g. *BAX*). *EXOC7* AS switches have been previously reported to occur during EMT in breast cancer [43]; one isoform containing an alternative 3´ region of exon 8 (isoform 5 or E) promotes a non-invasive epithelial phenotype, while another isoform lacking this region (isoform 2 or M) has been associated with a mesenchymal aggressive phenotype [43]. Herein, we have uncovered a novel *EXOC7* AS switch occurring in breast tumorigenesis. We found that a long isoform *EXOC7-L*, including the exon 7, is only present in MCF7 and MDA-MB-231 cell lines, whereas the short isoform *EXOC7-S*, lacking exon 7, is present in both MCF10-A and MCF7. Such AS switching has been also reported to occur in human fibroblasts [62]. Strikingly, our results showed a decrease of exon 7 inclusion of *EXOC7* upon silencing of *METTL3*. Additionally, we report that this event of *EXOC7* isoform switch has a prognosis value for breast cancer patients.

METTL3-regulated AS is not limited to m^6^A deposition at intronic regions. In addition to *cis*-acting RNA elements, dysregulated expression of splicing factors and their mediated splicing events are widely acknowledged to generate distinct AS events. Hence, our analysis revealed differential expression of spliceosome subcomplex components across MCF10-A, MCF7 and MDA-MB-231 cell lines. We further found that several mRNAs encoding splicing factors were decorated with m^6^A. Yet, although these transcripts harbor m^6^A, no major change at the mRNA level was found after silencing of *METTL3*. m^6^A in mRNA is known to primarily affect export, splicing, RNA stability, and translation, and therefore, m^6^A-mediated control of gene expression might not be reflected by changes in steady-state mRNA levels assessed by RNA-seq. One possible mechanism by which METTL3 can potentially regulate AS is *via* the proto-oncogene MYC as: i) it is implicated in AS in breast cancer [56]; ii) *MYC* mRNA is decorated with the m^6^A mark which positively regulates MYC expression; and iii) METTL3-mediated DSE significantly overlapped with MYC-regulated DSE, although we cannot disregard the possibility that those AS events are more sensitive to switches upon perturbation of the splicing factor that regulates them. Indeed the expression of multiple splicing factors, including members of the SF3A/3B complexes and SR proteins, correlate with MYC to control the pre-spliceosome assembly [11, 63]. Interestingly, MYC would not only regulate the expression of those splicing factors transcriptionally but also translationally. For instance, it has been recently described that the translation of SF3A3 is upregulated upon MYC hyperactivation [11]. Our data showed that METTL3 inhibition leads to decreased SRSF11 and MYC protein levels, suggesting that METTL3 may indirectly modulate AS. Additionally, it would be interesting to study the co-expression of SRSF11 with other splicing factors because even though the dysregulation of a single splicing factor can promote breast tumorigeneis and metastasis [39, 58], not all splicing factors can trigger tumorigenesis when expressed alone [64]. Therefore the potential dysregulation of other splicing factors and their subsequent splicing events still need to be explored, and this information might be particularly valuable to deepen our understanding of the biological relevance of METTL3 in breast cancer.

In this study, we found a higher PSI value in breast cancer patients than in normal samples for *COMMD4_AS2*, *GNAS*, *MATR3, RHOC and COMMD4_AS1*, whereas the PSI value was lower for *MARK3*, *POLDIP3* and FASTK. Nonetheless, we observed no differences in the case of *BAX* and *EXOC7*. These results did not fully reflect our findings from the DSE validated in the non-tumorigenic and the breast cancer cell lines in vitro. One possible explanation is that the AS events database gathers information of all breast cancer subtypes and each subtype is associated with a unique AS signature. However, we cannot rule out the possibility that other factors influence the apparent differences between cell lines and patients, a key challenge for translating findings to the clinic. Despite that the AS events analyzed have been previously described in breast cancer and other types of cancer, we have observed variabilities in the change of the PSI value along the progression of breast cancer. For instance, *EXOC7* and *FASTK* displayed a lower PSI at stage III and an increase at stage IV, while for *POLDIP3* the PSI increased at stage III but decreased at stage IV. This indicates that metastasis evolving from a primary tumor is a complex process whereby the tumor acquires metastatic characteristics through additional variables. Additionally, further studies should address whether the difference between our validation in vitro and the TCGA SpliceSeq analysis could arise from the cancer heterogeneity in patients, or whether the cancers originate from a single progenitor cell or from polyclonal seeding, leading to different outcomes during tumorigenesis [65, 66]. Moreover, supporting our results, previous studies have shown genetic differences between primary tumors and lymph node metastases [67–69], because cells can evolve independently of the primary tumor and that different tumor clones can be seeded in parallel to distant sites.

In summary, our study provides further insight into the function of METTL3 and m^6^A in breast cancer by regulating tumor-associated AS switches. Future work should uncover whether these DSE result directly from m^6^A deposition at splice sites or arise from a dysregulated expression of splicing factors, and provide new insights into the regulation and function of m^6^A-associated AS within individual cells from a given population. A better understanding of these molecular mechanisms will then potentially improve the therapeutic opportunities that specifically target breast cancer-associated AS isoforms.

## Materials and methods

### Antibodies

The following commercially available antibodies were used at the indicated concentrations for western blot: Anti-METTL3 (Abcam, ab221795, 1:5 000), Anti-METTL5 (Proteintech, 1:1 000), Anti-MYC (Thermo 13-2500, 1:2 000), Anti-SRSF11 (Abcam, ab196801), Anti-Actin (Sigma, A5441, 1:5 000), Anti-HDAC1 (Abcam, ab19845, 1:1 000), Goat Anti-Mouse IgG H&L (HRP) (Abcam, ab6789 1:10 000), Goat Anti-Rabbit IgG H&L (HRP) (Abcam, ab6721, 1:10 000).

### Cell culture

HEK293T, SKBR3, MCF7, MDA-MB-453, MDA-MB-231, and Hs578T cell lines were cultured in Dulbecco’s Modified Eagle Medium (DMEM, Gibco) supplemented with 10% fetal bovine serum (FBS, Gibco), and 1% penicillin/streptomycin (Gibco). For MCF7, T47D1, Hs578T and MDA-MB-231, media was additionally supplemented with 10 µg/ml human insulin (Sigma-Aldrich). T47D1 cell line was cultured in RPMI 1640 (Gibco) supplemented with 10% FBS and 10 µg/ml human insulin (Sigma). MCF10-A and hTERT-HME1 cell lines were cultured in DMEM/F12 (Sigma-Aldrich) supplemented with 5% heat-inactivated horse serum (Gibco), 20 ng/ml epidermal growth factor (Sigma-Aldrich), 0.5 mg/ml hydrocortisone (Sigma-Aldrich), 100 ng/ml cholera toxin (Sigma-Aldrich), 10 µg/ml insulin (Sigma-Aldrich), and 1% penicillin/streptomycin (Gibco). Cells were cultured at 37 °C in a humidified incubator at 5% CO_2_.

### Lentiviruses production and generation of *METTL3* knockdown cell lines

To generate lentiviral particles, HEK293T cells were co-transfected with pLKO.1-Puro containing shRNA1, shRNA2 against *METTL3* or scramble control, the packaging vector pCMV-dR8.2-dvpr and the envelope vector pCMV-VSV-G (ratio 6:8:2), with Jet-PEI Polyplus following the manufacturer’s instructions. Lentiviral particles were collected after 48 and 72 h, filtered through a 0.45 µm filter and concentrated using Amicon Ultra-15 Centrifugal Filter (Merck). Knockdown of *METTL3* was obtained by lentiviral transduction with the lentiviral particles in media supplemented with Polybrene (8 µg/ml). Transduced cells were selected by supplementing the culture media with puromycin (1 µg/ml) for an additional 4 days. The efficiency of *METTL3* knockdown was further evaluated by RT-qPCR and western blot analysis. All shRNA sequences are provided in Supplementary Table 5.

### Cellular proliferation

100 000 cells were seeded in 6-well plate and were counted using trypan-blue (Bio-Rad) every second day for 6 days.

### Apoptosis assay

Apoptosis assay was performed using a Muse Cell Analyzer (Millipore, Sigma-Aldrich) following the manufacturer’s instructions.

### Colony formation assay

10 000 cells were seeded in 6-well plate, and after 7 days, cell forming colonies were washed with PBS and stained with 0.3% crystal violet (Sigma-Aldrich) in methanol (ThermoFisher Scientific) for 20 min at room temperature. Colonies were washed 5 times with PBS and scanned for imaging.

### Reverse transcription followed by PCR (RT-PCR) and quantitative PCR (RT-qPCR)

Total RNA was extracted using the RNeasy Mini Kit (Qiagen) following the manufacturer’s recommendations. 1 μg of total RNA was reverse transcribed into cDNA using the RevertAid First Strand cDNA Synthesis kit (Invitrogen). Afterwards, PCR was performed using DreamTaq master mix (Thermo Fisher Scientific) for RT-PCRs. Quantitative PCR (qPCR) was performed using the Power Up SYBR Green qPCR Master Mix (Applied Biosystems) using an Agilent Biosystems instrument. *GAPDH* and *βactin* were used as loading control for RT-PCRs and RT-qPCRs, respectively. Primers are described in Supplementary Table 5.

### mRNA purification

mRNA was purified using Dynabeads^TM^ following the manufacturer’s recommendations. mRNA was eluted twice with RNase-free water.

### mRNA mass spectrometry analysis

Purified mRNA (100 ng) was analyzed by liquid chromatography-tandem mass spectrometry (LC-MS/MS) at the Proteomics and Modomics core facility, Norwegian University of Science and Technology (NTNU), Norway.

### RNA immunoprecipitation of m^6^A modified transcripts (MeRIP)

m^6^A modified transcripts were immunoprecipitated as described previously [70]. Briefly, 5 µg of mRNA was fragmented by using RNA fragmentation reagents (Invitrogen) prior to overnight ethanol precipitation. The fragmented mRNA was recovered by centrifugation at 14 000 rpm and the pellets were resuspended in DEPC water and 10% of the volume used as the input. The remaining fragmented mRNA was then diluted with 100 µl of 5× IP buffer (250 mM Tris pH 7.4, 500 mM NaCl, 0.25% NP-40) and incubated with 10 µg of m^6^A antibody (Abcam, ab151230) in the presence of RNase inhibitors, for 3 h at 4 °C. 30 µl of prewashed Surebeads Protein A magnetic beads (Bio-Rad) were added and incubated for 2 h at 4 °C. Beads were then washed twice with high-salt IP buffer (50 mM Tris pH 7.4, 1 M NaCl, 1 mM EDTA, 1% NP-40), twice with 1× IP buffer and finally once with high-salt IP buffer. The immunoprecipitated RNA was eluted in PK buffer (100 mM Tris-HCl pH 7.5, 50 mM NaCl, 10 mM EDTA) in the presence of Proteinase K (Invitrogen) recovered with Phenol:Chloroform. The input RNA and the immunoprecipitated RNA were subjected to reverse transcription using the VILO Superscript (Invitrogen™) according to the manufacturer’s instructions, followed by qPCR. Primers used for RT-qPCRs are described in Supplementary Table 5.

### PAR-CLIP

MDA-MB-231 stable cell line expressing Tet-off Flag-METTL3 was incubated with or without Doxycycline for 48 h. Cells were grown in the presence of 200 µM 4SU (Sigma Aldrich) for 14 h and were crosslinked (365 nm, 0.4 J cm^−2^). Cells were harvested and lysed with 1× NP lysis buffer (50 mM Tris HCl pH 7.5, 100 mM NaCl, 0.5% (v/v) NP-40, 2 mM EDTA, protease inhibitor cocktail (Thermo Fisher) and RNase inhibitor (Thermo Fisher) with gentle rotation for 30 min at 4 °C. The lysate was treated with RNAse T1 at 22 °C for 20 min followed by incubation on ice for 5 min. FLAG-METTL3 immunoprecipitation was carried out using Flag magnetic beads for 3 h at 4 °C with gentle rotation. Beads were washed 3 times with IP washing buffer (50 mM Tris-HCl pH 7.5, 300 mM NaCl, 0.05% (v/v) NP-40). Beads were resuspended in IP washing buffer supplemented with 20 U/µl of RNase T1 and incubated for 20 min at 22 °C followed by incubation on ice for 5 min. Beads were washed 3 times with high salt washing buffer (50 mM HEPES-KOH pH 7.5, 500 mM NaCl, 0.05% (v/v) NP-40) and once with Dephosphorylation buffer (50 mM Tris-HCl pH 7.9, 100 mM NaCl, 10 mM MgCl_2_). Beads were resuspended in Dephosphorylation buffer, and 10 U/µl Calf Intestinal alkaline phosphatase was added and incubated for 10 min at 37 °C. Beads were washed twice with Phosphatase washing buffer (50 mM Tris-HCl pH 7.5, 20 mM EGTA, 0.5% (v/v) Triton-X-100) and twice with PNK buffer (50 mM Tris-HCl pH 7.5, 50 mM NaCl, 10 mM MgCl_2_). After washing, beads were mixed with 5´ phosphorylation buffer (1× PNK buffer, ATP, T4 PNK enzyme and RNase Inhibitor) and incubated at 37 °C with rotation for 15 min and were washed 3 times with PNK buffer. 1/10 of beads is used for biotin labeling assay and the remaining is used for RNA extraction. For RT-qPCR analysis, samples were incubated with Proteinase K and RNA was extracted using Trizol method. RNA was reverse transcribed using SuperScript™ VILO™ cDNA Synthesis Kit (Thermo Fisher). Primers used for RT-qPCR are indicated in Supplementary Table 5.

### SELECT

SELECT was performed as previously described [55]. Briefly, 1 μg of total RNA from MDA-MB-231 cells treated with STM2457 or DMSO (control) was diluted in 5 μM dNTP, 1× CutSmart buffer (NEB), 40 nM up- and 40 nM down-primers. The primers are specific to an m^6^A site or to a control sequence (referred as input) located upstream of the m^6^A site. Annealing of primers was done at 90 °C, 1 min; 80 °C, 1 min; 70 °C, 1 min; 60 °C, 1 min; 50 °C, 1 min; 40 °C, 6 min. Ligation was performed in presence of 0.01 U Bst2.0 DNA Polymerase (NEB), 0.5 U SplintR ligase (NEB), 10 nmol ATP and incubated at 40 °C for 20 min then at 80 °C for 20 min. qPCR was further performed using 6 μl of the reaction products. Relative SELECT products were normalized by the input and the control cells (DMSO). All primers sequences are provided in Supplementary Table 5.

### mRNA stability assay

Cells were treated with 5 µg/ml actinomycin D (Sigma) and collected at the indicated time points. Total RNA was extracted as previously described and *MYC* mRNA level was assessed by RT-qPCR to determine its turnover rate.

### Reporter cloning and luciferase assay in MDA-MB-231 cell line

All primers and sequences are provided in Supplementary Table 5. The wild type sequence of *MYC* 3´UTR was amplified by PCR (Phusion™ High-Fidelity DNA Polymerase, Thermo Scientific™) from cDNA obtained from MCF10-A cell line using the RevertAid kit (Thermo Fisher) with oligo(dT) primers. The sequence was digested using *XhoI* and *NotI* and inserted into the multiple cloning site of psiCheck2 plasmid [71], which was digested with the same restriction enzymes beforehand and purified by QIAquick gel extraction kit (Qiagen). *MYC* 3´UTR sequence was validated by Sanger sequencing. For the luciferase assay, MDA-MB-231 cells were seeded in 24-well plates and transfected with *MYC* 3´UTR reporter plasmid or empty vector for control cells using lipofectamine LTX following the manufacturer’s instructions (Thermo Fisher). Cells were treated with METTL3 inhibitor STM2457 or DMSO (control), 24 h after transfection. The luciferase assay was carried out 48 h after STM2457 treatment, using Dual-Luciferase® Reporter Assay System (Promega) according to the manufacturer’s instructions. Data was normalized as the value of Renilla divided by Firefly luciferase; cells transfected with the empty vector and non-treated were set as 1.

### Immunoblotting

To assess protein levels, cells were prepared using cell lysis buffer containing 50 mM HEPES pH 7.5, 150 mM NaCl, 3 mM MgCl_2_, 0.2% Triton X-100, 0.2% Nonidet NP-40, 10% glycerol, protease inhibitor. Lysates were subjected to SDS-PAGE and transferred to PVDF membranes using wet transfer. Membranes were incubated in 5% skim milk in PBS-T (1× PBS, 0.1% Tween-20) for 1 h at room temperature and incubated with primary antibody (as describe above). The membrane was washed with PBS-T (0.1% Tween-20) three times for 5 min and incubated with secondary antibody (as describe above) diluted in PBS-T (0.1% Tween-20) for 1 h at room temperature. Protein detection was performed using Pierce™ ECL Western Blotting Substrate (Thermo Fisher) with Amersham AI680 imager.

### RNA-seq and differential gene expression analysis

RNA-seq library preparation was carried out at Novogene facilities (https://en.novogene.com/) and sequenced using Illumina HiSeq 2500 platform (Illumina) as 150 bp pair-ended reads. FASTQ reads were pseudoaligned to the human hg38 transcriptome and quantified using Salmon [72]. Thereafter, differentially expressed genes (DEG) were obtained using a MATLAB function with a test under the assumption of a negative binomial distribution where the variance is linked to the mean *via* a locally-regressed smooth function of the mean [73]. Afterwards, *P*-values were adjusted by estimation of the false discovery rate for multiple hypotheses [74]. We only considered the transcripts with reads in at least half of the samples analyzed.

### AS analysis using RNA-seq

To quantify the AS differences between sets of samples we employed the SUPPA2 pipeline [37]. Specifically, the Salmon output files generated for the RNA-seq were adapted for the SUPPA2. Splicing events in the human genome were obtained using a specific SUPPA2 script from the human GTF genome hg38 file. Thereafter, the percentage of splicing inclusion (PSI) values for each event were obtained for each sample, and the differential PSI values (ΔPSI) for each condition was calculated along with a *P-value* for each event. Ad hoc MATLAB functions were designed to quantify and represent the different analyses from the final SUPPA2 output files. In the case of publicly available datasets (MYC: GSE196325), the same pipeline from FASTQ reads was performed.

### m^6^A and PTC data analysis from public datasets and comparison with AS

The different m^6^A datasets used in the studies (MCF7: GSE143441; MDA-MB-231: GSM5616175; HEK293T: GSE114543) were standardized for comparison. Specifically, they were converted into hg38 and BED format and then subjected to MACS2 for peak detection [75]. Afterwards, we compared their results against AS (exon skipping) datasets by a set of scripts that require PERL and Bedtools [76]. Fisher’s exact test was applied to assess the statistical significance for the presence of intronic m^6^A sites in significantly spliced exons compared to non-significantly spliced genes. In the case of premature termination codons (PTCs), the splicing events in transcripts annotated as nonsense-mediated decay were analyzed by Fisher’s exact test in a similar manner than in the case of m^6^A.

### De novo motif search

m^6^A peaks that were located within flanking introns of a differentially skipped exon were selected. Then the sequence (+/−150 nt) of these peaks was submitted to de novo motif search using HOMER [77]. Afterwards, random genomic regions with similar properties of these peaks were retrieved for direct comparison of density distribution along the m^6^A region.

### Gene Ontology (GO) analysis

Gene ontology (GO) analysis was performed using the web tool The Database for Annotation, Visualization and Integrated Discovery (DAVID) [78] (https://david.ncifcrf.gov/).

### Analysis of TCGA datasets using SpliceSeq database

A set of validated splicing events was selected and its PSI data retrieved in breast cancer datasets from TCGA using SpliceSeq [56]. The cBioportal webserver was used for obtaining METTL3 gene expression as well as Copy Number Variation (CNV) of its locus in the same breast cancer samples, and the clinical data associated to these samples was obtained from TCGA. Briefly, MATLAB functions were designed to calculate new coefficients for each event using the Lasso function, using the 75^th^ percentil of the risk score as cutoff for the classification of patients in high (302 patients) and low risk (905 patients). In addition, a combined splicing signature was obtained for a set of AS events using the combined coefficients from the Lasso risk function in a multivariate Cox analysis. The formula for the calculation of risk score for each patient was calculated as (β_AS event 1_ × PSI_AS event 1_) + (β_AS event 2_ × PSI_AS event 2_) + … + (β_AS event n_ × PSI_AS event n_), as previously described [79]. Finally, we generated box plots, as well as Kaplan–Meier survival curves using MATLAB; statistical *P-values* for every event and associated feature were also calculated, and R values were obtained for the correlations between *METTL3* gene expression and AS PSIs.

### Statistical analysis

Data are shown as mean ± SEM. GraphPad Prism version 8.0.0 was used to perform the statistical analysis. The significance was determined using Student’s *t* test, one-way and two-way ANOVA. Probability values of *P-valu*e* < 0.05, **P-value < 0.01, ***P-value < 0.001, ****P-value < 0.0001 were considered as statistically significant.

## Supplementary information

Supplementary Information

Supplementary Table 1

Supplementary Table 3

Supplementary Table 4

## Data Availability

All next-generation sequencing data can be publicly accessed in ArrayExpress webserver (E-MTAB-11664).
