# Supplementary material for: METTL3 regulates breast cancer-associated alternative splicing switches
Source: Oncogene. 2023 Feb 1;42(12):911–25. doi: 10.1038/s41388-023-02602-z (PMC10020087; doi:10.1038/s41388-023-02602-z)
Supplement: Supplementary file 1 — Supplementary Information [file 41388_2023_2602_MOESM1_ESM.docx]

**Supplementary Information**

**METTL3 regulates breast cancer-associated alternative splicing switches**

Cyrinne Achour^1,2^, Devi Prasad Bhattarai^1,2^, Paula Groza^1,2^, Ángel-Carlos Román^3^*, Francesca Aguilo^1,2^*

**Index of Supplemental Figures:**

- **Supplemental Figure 1.** Identification of DSE in breast cancer cell lines.
- **Supplemental Figure 2.** Characterization of METTL3 in breast cancer cell lines.
- **Supplemental Figure 3.** Genome-wide analysis of METTL3-mediated AS.
- **Supplemental Figure 4.** METTL3 mediates AS *via* m^6^A deposition.
- **Supplemental Figure 5.** m^6^A regulates MYC-associated AS events.
- **Supplemental Figure 6.** DSE signature in breast cancer patients.
- **Supplemental Figure 7.** Correlation between *METTL3* expression and AS events.

**Index of Supplemental Tables:**

- **Supplemental Table 1 (provided as a single Excel spreadsheet).** List of the DSE in the breast cancer cell lines MCF7 and MDA-MB-231 compared to the non-tumorigenic cell line MCF10-A.
- **Supplemental Table 2.** Function in cancer and type of altered AS event for the transcripts assessed in this study.
- **Supplemental Table 3 (provided as a single Excel spreadsheet).** List of the differentially expressed genes upon knockdown of *METTL3* in MCF10-A, MCF7 and MDA-MB-231.
- **Supplemental Table 4 (provided as a single Excel spreadsheet).** List of the DSE upon *METTL3* knockdown in MCF7 and MDA-MB-231 cell lines.
- **Supplemental Table 5.** Primers and shRNAs sequences used in this study.

**Supplemental Figure 1**

**
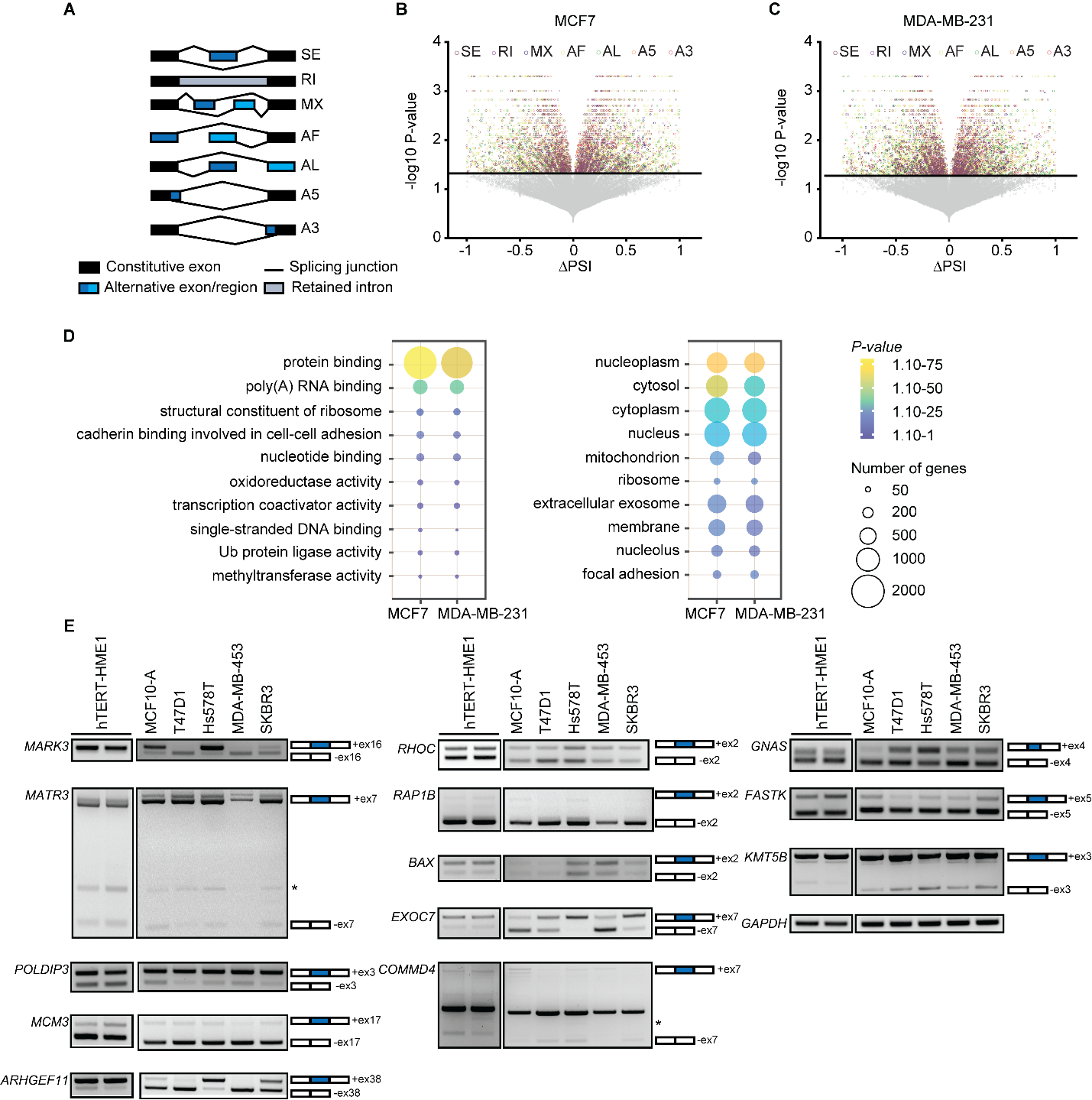
**

*Figure legend in the next page.*

**Supplemental Figure 1.** Identification of differential AS events (DSE) in breast cancer cell lines. **(A)** Schematics of the seven types of AS events. SE: skipped exon; RI: retained intron; MX: mutually exclusive exon; AF: alternative first exon; AL: alternative last exon; A5: 5´splice site; A3: 3´splice site. **(B-C)** Volcano plots representing the ∆PSI of the differentially spliced genes in MCF7 and MDA-MB-231 related to MCF10-A cells. The significant DSE are shown in a color code. The y-axis represents the –log10(*P-value)* where *P-value*<0.05. **(D)** Dot plots representing the Gene Ontology (GO) enrichment analysis of the common AS genes in MCF7 and MDA-MB-231. CC: cellular component, MF: molecular function. The size and the color of the dots are proportional to the number of genes enriched in each GO term and the significance of the enrichment (1.10^-75^<*P-value*<1.10^-1^), respectively. **(E)** RT-PCR indicating the different splicing events between the non-tumorigenic hTERT-HME1 and MCF10-A, and a panel of breast cancer cell lines T47D1, Hs578T, MDA-MB-453 and SKBR3. The annotation numbers of the skipped exons are depicted for each splicing events. The PSI was calculated in percentage for each gene. Non-specific bands are indicated with an asterisk.

**Supplemental Figure 2**

**
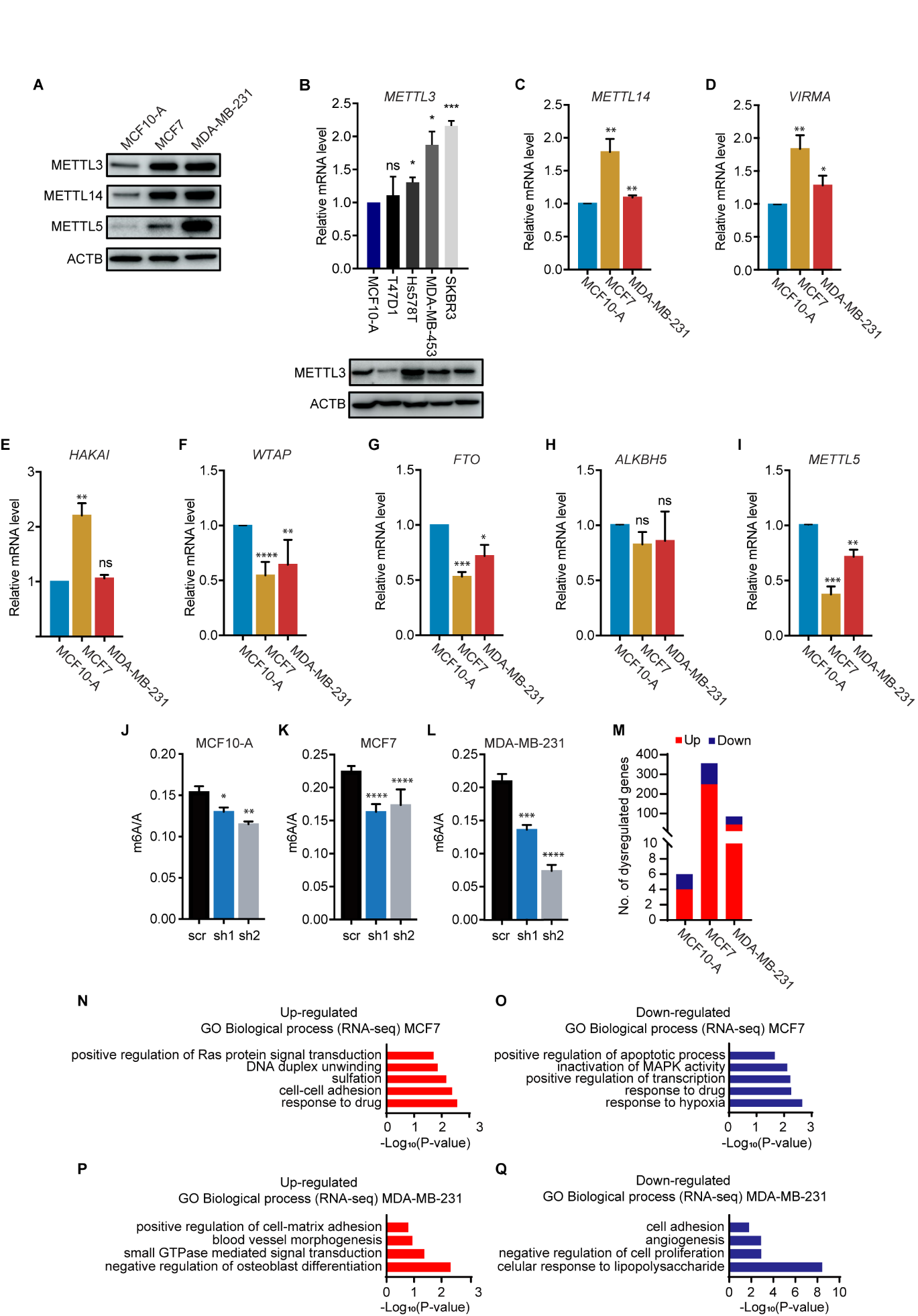
**

*Figure legend in the next page.*

**Supplemental Figure 2.** Characterization of METTL3 in breast cancer cell lines. **(A)** Western blot of METTL3, METTL14 and METTL5 in whole cell extracts (WCE) from MCF10-A, MCF7 and MDA-MB-231 cell lines. β-ACTIN (ACTB) is used as the loading control. **(B)** RT-qPCR analysis of *METTL3* mRNA levels in MCF10-A and the breast cancer cell lines T47D1, Hs578T, MDA-MB-453 and SKBR3. All transcripts are normalized to *β-ACTIN*. *P-values* were determined by two-tailed *t*-test. Western blot of METTL3 in WCE from MCF10-A, T47D1, Hs578T, MDA-MB-453 and SKBR3. β-ACTIN (ACTB) is used as the loading control. **(C-I)** RT-qPCR analysis of *METTL14, VIRMA, HAKAI, WTAP, FTO, ALKBH5* and *METTL5* mRNA levels in the normal mammary epithelial cell line MCF10-A and the breast cancer cell lines MCF7 and MDA-MB-231. All transcripts are normalized to *β-ACTIN*. *P-values* were determined by two-tailed *t*-test. **(J-L)** LC-MS/MS quantification of m^6^A in mRNA of **(J)** MCF10-A, **(K)** MCF7 and **(L)** MDA-MB-231 cells upon depletion of METTL3. Methylated adenosines are normalized to the total of unmodified adenosines. *P-values* were determined by two-way ANOVA. **(M)** Number of common up- (red) and down-regulated (blue) genes in MCF10-A, MCF7 and MDA-MB-231 cells upon depletion of METTL3. **(N-Q)** Gene ontology (GO) analysis of the biological processes associated to the **(N)** up- (red) and **(O)** down-regulated (blue) genes for MCF7 upon depletion of METTL3. **(P-Q)** Gene ontology (GO) analysis of the biological processes associated to the **(P)** up- (red) and **(Q)** down-regulated (blue) genes for MDA-MB-231 upon depletion of METTL3. Data in panels B-K are mean ± SEM of three independent experiments; **** p<0.0001, ** p<0.01, * p<0.05, ns non-significant.

**Supplemental Figure 3**

**
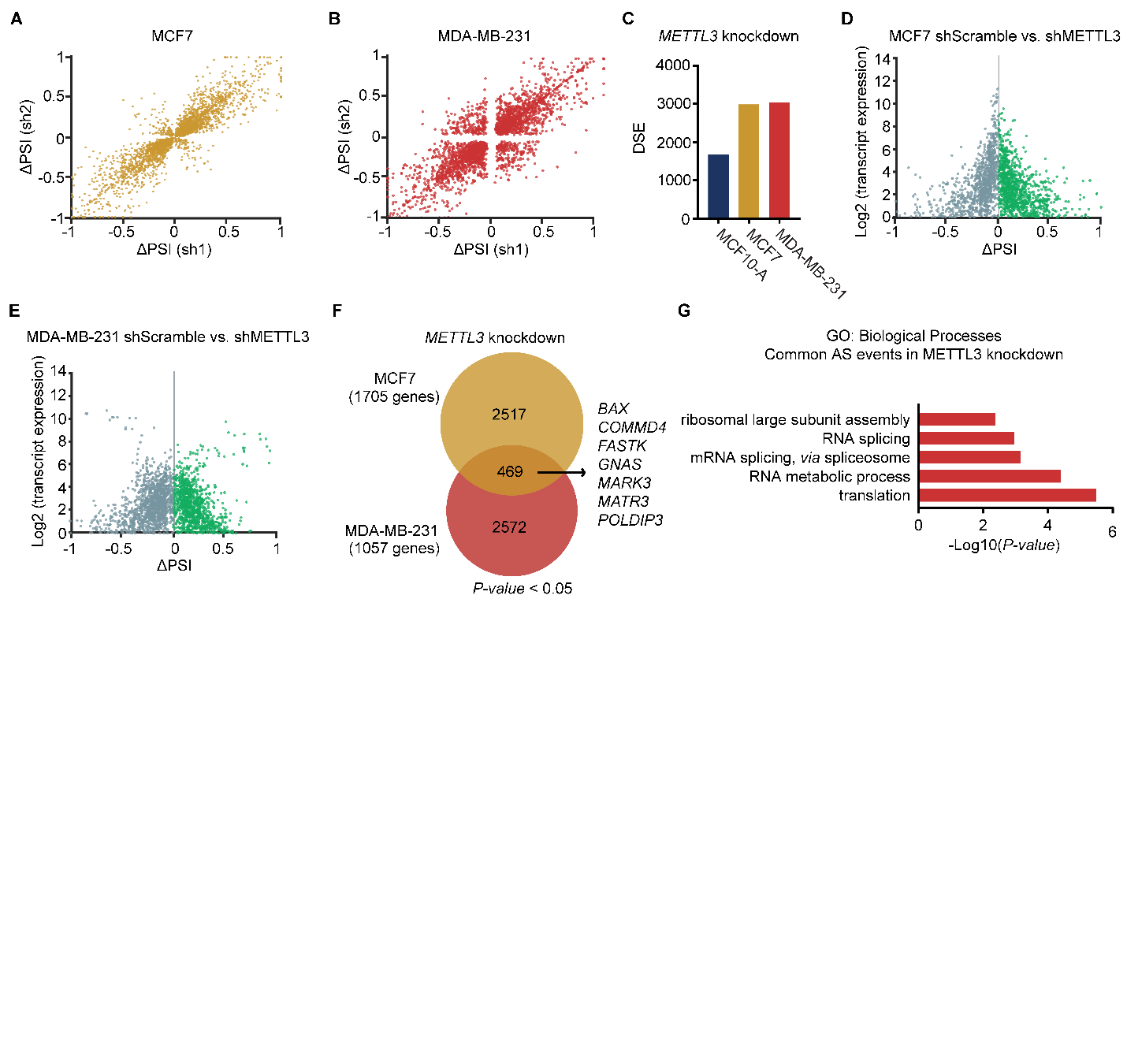
**

**Supplemental Figure 3.** Genome-wide analysis of METTL3-mediated AS. **(A-B)** Correlation of ∆PSI between biological replicates upon silencing of *METTL3* (sh1 and sh2) in MCF7 and in MDA-MB-231 cells. **(C)** Number of DSE in the non-tumorigenic cell line MCF10-A and the breast cancer cell lines MCF7 and MDA-MB-231 upon silencing of *METTL3*. **(D-E)** Volcano plot showing the correlation between gene expression levels and ∆PSI resulted from RNA-seq data analysis in control and METTL3 depleted MCF7 and MDA-MB-231 cell lines. **(F)** Venn diagrams depicting the common DSE between MCF7 (yellow) and MDA-MB-231 (red) upon depletion of METTL3. The number of DSE for each cell line is indicated in brackets; *P-value*<0.05. **(G)** GO analysis of common AS genes between MCF7 and MDA-MB-231 upon depletion of METTL3.

**Supplemental Figure 4**

**
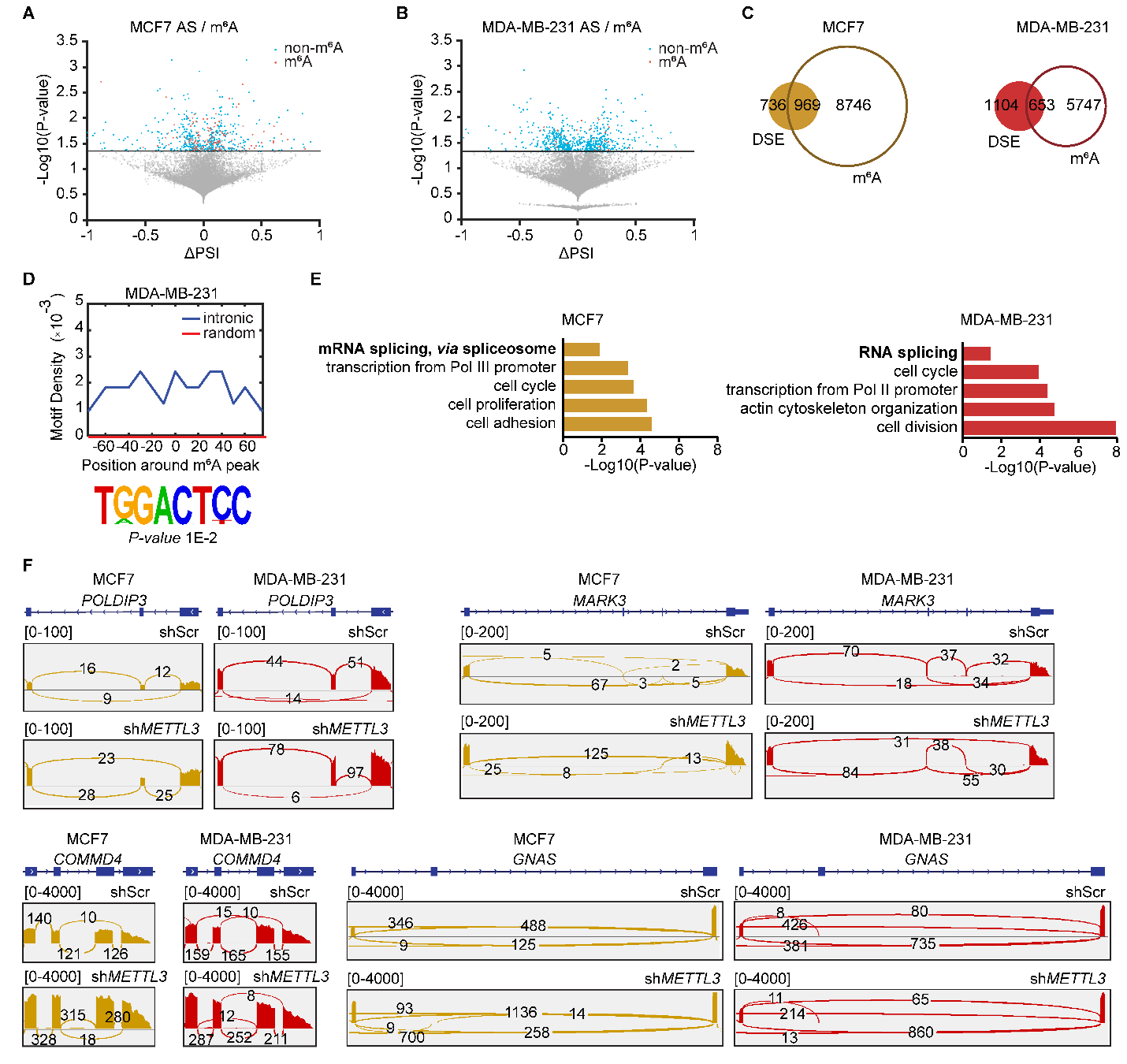
**

*Figure legend in the next page.*

**Supplemental Figure 4.** METTL3 mediates AS *via* m^6^A deposition. **(A-B)** Volcano plot of the ∆PSI of the differentially spliced transcripts in MCF7 and in MDA-MB-231. Highlighted in red are the transcripts harboring m^6^A and in blue the non-m^6^A modified transcripts (*P-value*<0.05). Grey dots indicate non-significant DSE. Datasets from (1); datasets GEO accession number for MCF7: GSE143441 and MDA-MB-231: GSM5616175. **(C)** Venn diagrams depicting the DSE harboring m^6^A mark in knockdown of *METTL3* in MCF7 and MDA-MB-231 cell line. **(D)** DRACH motif density (lowly represented; *P-value* 0.01) of m^6^A peaks in the –80 to +80 nt region around the m^6^A peak in intronic or random regions (left panel) and the corresponding HOMER motifs outputs (right panel) in MDA-MB-231. **(E)** GO analysis of biological processes associated to the m^6^A-modified transcripts in the breast tumorigenic cell lines MCF7 and MDA-MB-231 upon depletion of METTL3. *P-value*<0.05. **(F)** Examples of sashimi plots showing changes of AS events upon knockdown of *METTL3* in MCF7 and MDA-MB-231.

**Supplemental Figure 5**

**
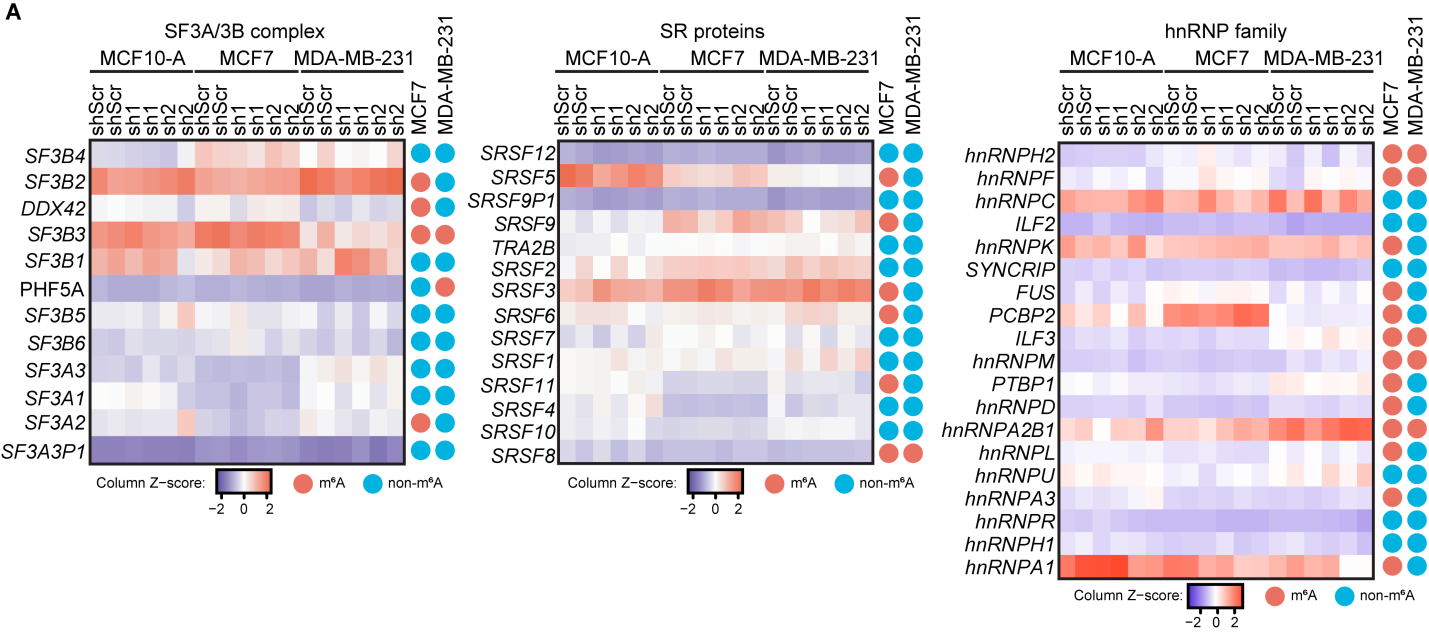
**

**Supplemental Figure 5.** m^6^A regulates MYC-associated AS events. **(A)** Heatmaps showing the differential expression for splicing factors within the SF3A/3B complex (left panel), SR proteins (middle panel) and hnRNP family of proteins (right panel) in MCF10-A, MCF7 and MDA-MB-231 cell lines. For each transcript encoding for a splicing factor, is indicated whether it is m^6^A modified (red dot) or non-m^6^A modified (blue dot). Heatmaps were scaled by row. The heatmaps were scaled with Z-Score using the Log_2_(FPKM) expression.

**Supplemental Figure 6**

**
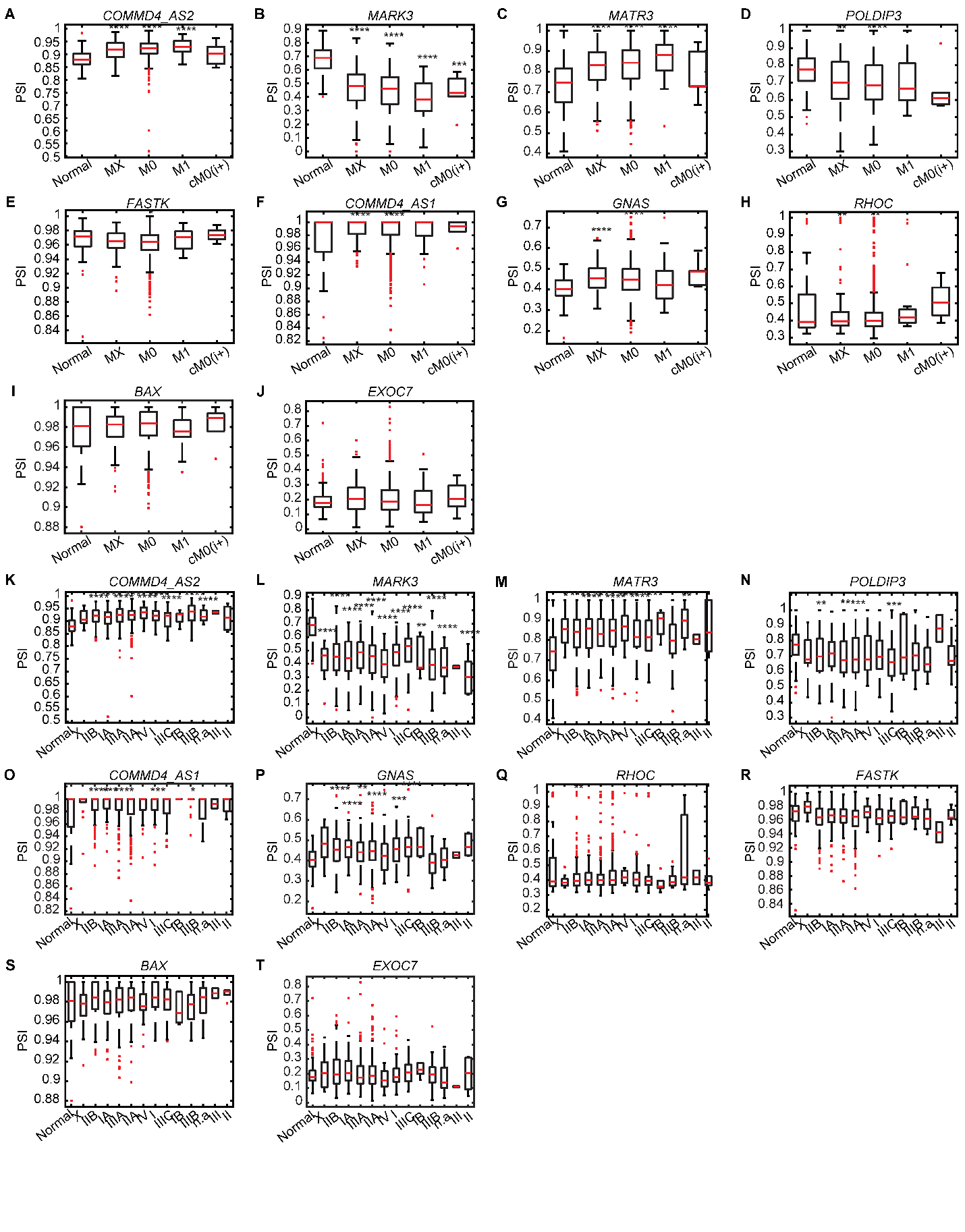
**

*Figure legend in the next page*

**Supplemental Figure 6.** DSE signature in breast cancer patients. **(A-J)** Box plots representative of the PSI values across breast tumor grades and in normal samples. M0: no distant metastasis, M1: distant metastasis, MX: distant metastasis cannot be assessed, cM0(i+): cancer cells present in blood or bone marrow or in lymph nodes farther from the primary site. **** *P-value*<0.0001, *** *P-value*<0.001, ** *P-value*<0.01; * *P-value*<0.05. **(K-T)** Box plots representative of the PSI values across breast tumor stages and in normal samples. Stage I: 90 patients; stage IA: 86; stage IB: 6; stage II: 6; stage IIA: 358; stage IIB: 255; stage III: 2; stage IIIA: 154; stage IIIB: 27; stage IIIC: 65; stage IV: 20; stage X: 13; n.a. (no stage assessed): 11; **** *P-value*<0.0001, *** *P-value*<0.001, ** *P-value*<0.01; * *P-value*<0.05.

**Supplemental Figure 7**

**
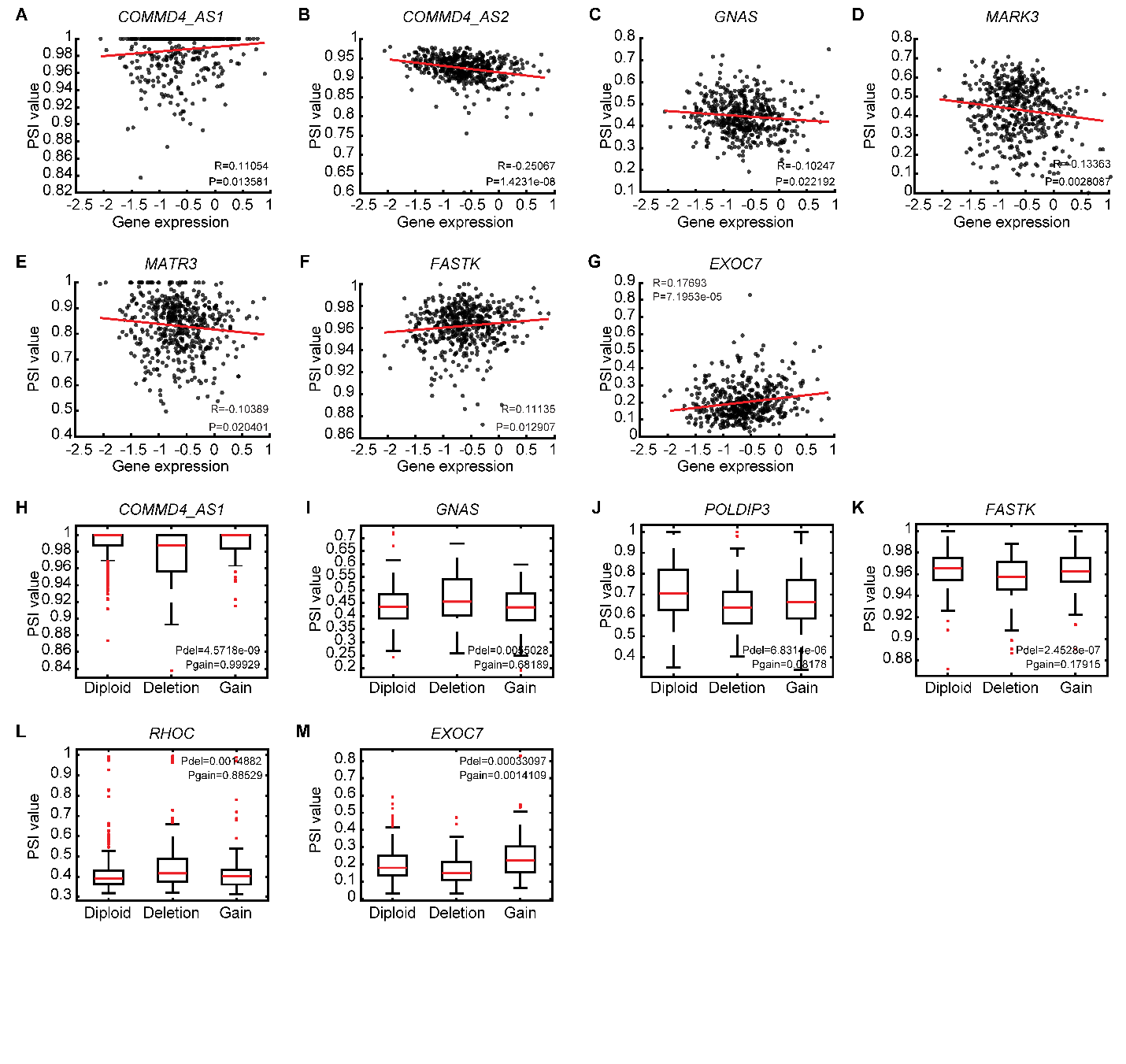
**

**Supplemental Figure 7.** Correlation between METTL3 expression and AS events. **(A-G)** Correlation between gene expression level of METTL3 and PSI values for the DSE identified in *COMMD4_AS1*, *FASTK*, *EXOC7* (positive correlation)*; COMMD4_AS2*, *GNAS*, *MARK3*, *MATR3* (negative correlation). Pearson correlation (R) values and *P-values* (P) are indicated within each graph. All *P-values*<0.05. **(H-M)** Box plots representing the association between the PSI values from the DSE identified in *COMMD4_AS1*, *GNAS*, *POLDIP3*, *FASTK*, *RHOC*, *EXOC7* and *METTL3* expression in breast cancer patients (deletion or gain). Diploid: normal expression of *METTL3*; deletion: downregulation of *METTL3*; gain: upregulation of *METTL3.* *P-values* (P) are indicated within each graph.

**Supplemental Table 2.** Function in cancer and type of altered AS event for the transcripts assessed in this study.

| **Gene name** | **Ensembl ID** | **Function** | **Type of AS events** | **Publication** |
| --- | --- | --- | --- | --- |
| *ARHGEF11* | ENSG00000132694 | EMT | SE exon 38 | (2) |
|  |  |  | SE | (3) |
|  |  |  | A3'SS, RI, A5'SS, SE | (4) |
|  |  |  | SE | (5) |
|  |  |  | SE | (6) |
| *BAX* | ENSG00000087088 | Apoptosis |  | (7) |
|  |  |  | A3'SS,RI | (4) |
|  |  |  | SE | (8) |
|  |  |  | SE | (3) |
| *COMMD4* | ENSG00000140365 | Unknown | RI, SE | (4) |
| *EBPL* | ENSG00000123179 | Unknown | MXE | (6) |
| *EXOC7* | ENSG00000182473 | EMT, invasion | SE | (9) |
|  |  |  | SE | (3) |
|  |  |  | RI, A3'SS, A5'SS, SE | (4) |
|  |  |  | MXE | (6) |
| *FASTK* | ENSG00000164896 | Apoptosis | RI, A3'SS | (4) |
|  |  |  | RI intron 5 | (10) |
| *GNAS* | ENSG00000087460 | Signaling, proliferation, migration (EMT) | SE | (8) |
|  |  |  | SE | (4) |
| *KMT5B* | ENSG00000110066 | Migration, adhesion | SE exon 3, A5'SS, RI | (4) |
| *MARK3* | ENSG00000075413 | Unknown | SE, RI | (4) |
|  |  |  | SE exon 15, 16 | (8)  (11) |
|  |  |  | SE | (6) |
| *MATR3* | ENSG00000015479 | EMT, apoptosis |  | (10) |
|  |  |  | SE, A3'SS, A5'SS | (4) |
|  |  |  | SE | (8) |
|  |  |  | SE | (6) |
| *MCM3* | ENSG00000112118 | Proliferation, drug resistance | SE | (4) |
| *POLDIP3* | ENSG00000100227 | Unknown | SE exon 3 | (10) |
| *RAP1B* | ENSG00000127314 | Cell-cell adhesion | A3'SS, MXE, SE, A5'SS | (4) |
| *RHOC* | ENSG00000155366 | Cell-cell junction, interferon signaling | SE | (8) |
|  |  |  | A5'SS, A3'SS | (4) |

**Supplemental Table 5.** Primers, constructs and shRNAs sequences used in this study.

| **RT-PCR** | | |
| --- | --- | --- |
| **Gene** | **Forward primer 5´🡪 3´** | **Reverse primer 5´🡪 3´** |
| *ARHGEF11* | GGCAGCAGGAGGTTACAAAG | TGAGCCTGTTGAGCTTGAGA |
| *BAX* | TGATGGACGGGTCCG | GGCAAAGTAGAAAAGGGCGAC |
| *COMMD4* | GCGAATCCTTGTCCAGTGAA | TCCCGAAGAGCAGTTCAGAG |
| *EBPL* | TTTCCTCTGCCGCATGGT | AACAATGCCAGAGACCCATC |
| *EXOC7* | TGGCCGCAACCAAGATTTCATG | TCGGACAGGTGCTTAACTCGGAAAT |
| *FASTK* | CATCTTGATGTCACTGTGCCA | CAGCAGGGAGAGGTAGCG |
| *GNAS* | AAAAGCACCATTGTGAAGCA | TCAATCGCCTCTTTCAGGTT |
| *KMT5B* | CGGTTTTCGTACGGGCGG | AAATAGTGCCGTGCCCATTC |
| *MARK3* | AAGAGGCACTGCCAGTCGTA | GAGCCCTCATATCTCCCGTTC |
| *MATR3* | GCCTTCACCTGAATGACATCT | GACGACTGTGACTTGCTCCA |
| *MCM3* | TCCAAAGACGGCAGACTCAC | TCCTGCATCTTGCTCAGAGC |
| *POLDIP3* | TGCCTTCATAAACCCACCCA | CATGTGGTGGAGAAAGCCG |
| *RAP1B* | ACATCGCCAAACCTCGCC | CGTTCCTGCAGTATCCAAGA |
| *RHOC* | TCTGAGCCTCCGGCACC | GGACGTAGACCTCCGGAAAC |

| **qPCR** | | |
| --- | --- | --- |
| **Gene** | **Forward primer 5´🡪 3´** | **Reverse primer 5´🡪 3´** |
| *Β-ACTIN* | AGATCAAGGTGGGTGTCTTTC | AGCAATGATCTGAGGAGGGAAG |
| *GAPDH* | TGGTATCGTGGAAGGACTCA | CCAGTAGAGGCAGGGATGAT |
| *METTL3* | AACTGCAACGCATCATTCGG | TTGACACCAACCAAGCAGTG |
| *MYC* | CATCAGCACAACTACGCAGC | GCTGGTGCATTTTCGGTTGT |
| *METTL5* | GCCCAAGCTACTTCTGGAACAG | CCGATGCTAAGTACTCCACAACC |
| *METTL14* | AGGGGTTGGACCTTGGAAGA | GAAGTCCCCGTCTGTGCTAC |
| *WTAP* | AAGCAACAACAGCAGGAGTC | TTGATCTCAGTTGGGCAACG |
| *VIRMA* | CGATAACTTGATGACCCCAGAA | ATAACGGCAAGATTCCATTTC |
| *HAKAI* | TGTTACCCGTGCTTCACTTG | TCATGATGTGCTGCTTTGGC |
| *FTO* | TGTTTTGGCCGGTTCACAAC | ACATTCTGCAGAGCCAACTG |
| *ALKBH5* | TGCAAGTTCCAGTTCAAGCC | GCCGTATGCAGTGAGTGATTTC |

| **Primers for SELECT of *MYC* m^6^A site** | |
| --- | --- |
| *m^6^A site_up* | TAGCCAGTACCGTAGTGCGTGCCCTCTTGGCAGCAGGATAG |
| *m^6^A site_down* | CCTTCCGAGTGGAGGGAGGCCAGAGGCTGAGTCGCTGCAT |
| *Input_up* | TAGCCAGTACCGTAGTGCGTGTGGCAGCAGGATAGTCCTTC |
| *Input_down* | GAGTGGAGGGAGGCGCTGCGCAGAGGCTGAGTCGCTGCAT |
| *qPCR_SELECT_Forward* | ATGCAGCGACTCAGCCTCTG |
| *qPCR_SELECT_Reverse* | TAGCCAGTACCGTAGTGCGTG |

| **shRNAs** | | |
| --- | --- | --- |
| **Target Gene** | **Plasmid** | **Sequence 5´🡪 3´** |
| Scramble | pLKO.1-puro-shScramble | CAACAAGATGAAGAGCACCAA |
| *METTL3* | pLKO.1-puro-sh*METTL3*_1 | GCAAGTATGTTCACTATGAAA |
|  | pLKO.1-puro-sh*METTL3*_2 | CGTCAGTATCTTGGGCAAGTT |

| **Luciferase reporter assay** | |
| --- | --- |
| psiCHECK2 | All predicted m^6^A sites in *Renilla* and *Firefly Luciferase* were mutated. |
| XhoI-MYC_3´UTR-Forward | GTCGAGCATCAGCACAACTACGCAGC |
| NotI-MYC_3´UTR-Reverse | GCGGCCGCGAGGTTGCATTTGATCATGC |
| Sequencing primer | GCCTAAGATGTTCATCGAGTC |

LITERATURE CITED

1. Lee JH, Wang R, Xiong F, Krakowiak J, Liao Z, Nguyen PT, et al. Enhancer RNA m6A methylation facilitates transcriptional condensate formation and gene activation. Mol Cell. 2021;81(16):3368-85 e9.

2. Itoh M, Radisky DC, Hashiguchi M, Sugimoto H. The exon 38-containing ARHGEF11 splice isoform is differentially expressed and is required for migration and growth in invasive breast cancer cells. Oncotarget. 2017;8(54):92157-70.

3. Anczukow O, Akerman M, Clery A, Wu J, Shen C, Shirole NH, et al. SRSF1-Regulated Alternative Splicing in Breast Cancer. Mol Cell. 2015;60(1):105-17.

4. Park S, Brugiolo M, Akerman M, Das S, Urbanski L, Geier A, et al. Differential Functions of Splicing Factors in Mammary Transformation and Breast Cancer Metastasis. Cell Rep. 2019;29(9):2672-88 e7.

5. Gokmen-Polar Y, Gu Y, Gu X, Badve SS. Splicing factor ESRP1 controls ER-positive breast cancer progression by altering metabolic pathway genes. Cancer Research. 2019;79(4).

6. Shapiro IM, Cheng AW, Flytzanis NC, Balsamo M, Condeelis JS, Oktay MH, et al. An EMT-driven alternative splicing program occurs in human breast cancer and modulates cellular phenotype. PLoS Genet. 2011;7(8):e1002218.

7. Kholoussi NM, El-Nabi SEH, Esmaiel NN, Abd El-Bary NM, El-Kased AF. Evaluation of Bax and Bak Gene Mutations and Expression in Breast Cancer. Biomed Res Int. 2014;2014.

8. Oh J, Pradella D, Shao CW, Li HR, Choi N, Ha J, et al. Widespread Alternative Splicing Changes in Metastatic Breast Cancer Cells. Cells-Basel. 2021;10(4).

9. Lu HZ, Liu JL, Liu SJ, Zeng JW, Ding DQ, Carstens RP, et al. Exo70 Isoform Switching upon Epithelial-Mesenchymal Transition Mediates Cancer Cell Invasion. Dev Cell. 2013;27(5):560-73.

10. Zheng YZ, Xue MZ, Shen HJ, Li XG, Ma D, Gong Y, et al. PHF5A Epigenetically Inhibits Apoptosis to Promote Breast Cancer Progression. Cancer Res. 2018;78(12):3190-206.

11. Oh J, Pradella D, Kim Y, Shao C, Li H, Choi N, et al. Global Alternative Splicing Defects in Human Breast Cancer Cells. Cancers (Basel). 2021;13(12).
